# Constitutive Androstane Receptor in Macrophages Regulates Toll‐Like Receptor 4‐Mediated Innate Immune Responses Against Endotoxemic Liver Injury

**DOI:** 10.1002/advs.202506725

**Published:** 2025-08-18

**Authors:** Renjie Cao, Tingting Zhao, Ying Wang, Shaofei Song, Yuan Li, Mengling Hou, Yanxin Zhang, Sengpeng Wong, Siqi Wang, Yuran Wang, Hong Peng, Min Huang, Yiming Jiang

**Affiliations:** ^1^ Guangdong Provincial Key Laboratory of New Drug Design and Evaluation School of Pharmaceutical Sciences Sun Yat‐Sen University Institute of Clinical Pharmacology Sun Yat‐Sen University Guangzhou 510006 China; ^2^ Sun Yat‐Sen Memorial Hospital Sun Yat‐Sen University Guangzhou 510006 China; ^3^ Center of Hepato‐Pancreato‐biliary Surgery The First Affiliated Hospital of Sun Yat‐Sen University Guangzhou 510006 China

**Keywords:** constitutive androstane receptor, endotoxemia, liver injury, toll‐like receptor 4

## Abstract

Macrophage proinflammatory hyperactivation drives the pathogenesis of acute liver injury, a common complication of sepsis. The role of the nuclear receptor constitutive androstane receptor (CAR) in endotoxin‐induced liver injury remains unclear. Here, this study reports that CAR is highly expressed in human and murine macrophages. CAR activation markedly attenuated endotoxin‐induced liver damage, alleviating hepatocyte death and hepatic inflammation. Macrophage‐hepatocyte coculture confirmed that CAR inhibited inflammation through macrophage crosstalk. CAR‐mediated hepatoprotection and anti‐inflammatory effects are absent in AAV8‐*F4/80‐*sh*Car*‐treated mice, confirming the essential role of CAR in macrophages. Mechanistically, CAR is found to interact with *Tlr4*, and the suppressive effects of CAR on TLR4 are proven in *Tlr4* ^−/−^ mice. Furthermore, CAR activation reduced LPS‐induced inflammation in hMDMs, BMDMs, RAW264.7, and THP‐1 cells, and *Car* or *Tlr4* knockdown abolished CAR‐mediated immunosuppression. Overall, these findings showed that macrophage CAR activation attenuated endotoxin‐induced liver injury and hepatic inflammation through the TLR4 signaling pathway, providing insights for treating inflammatory liver diseases.

## Graphical abstract

The present study uncovers a novel role of constitutive androstane receptor (CAR) in inflammation‐mediated damage. CAR activation in macrophages inhibits proinflammatory macrophages by interacting with *Tlr4*, which protects against endotoxemic liver injury. CAR agonist may be provided as a promising therapeutic strategy for treating endotoxemic liver injury.

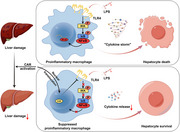

## 1 Introduction

Acute liver failure has a high mortality rate and a poor prognosis, and often occurs secondary to infection or a severe immune response.^[^ ^1^ ^]^ Endotoxemia is caused by microbial infection or bacterial products, such as lipopolysaccharide (LPS), an endotoxin from gram‐negative bacteria that is one of the most potent stimulators of the innate immune response.^[^ ^2^ ^]^ In endotoxemic patients, LPS release stimulates macrophages to secrete a variety of inflammatory factors and chemokines, such as interleukin 6 (IL6) and tumor necrosis factor α (TNFα), generating a “cytokine storm” that ultimately leads to liver injury.^[^ ^3^ ^]^ Despite its high rates of mortality and morbidity, there is no effective therapy for end‐stage liver failure other than liver transplantation. Therefore, exploring novel therapies to improve the overall survival of patients with endotoxin‐induced liver injury has become a challenge for clinicians.

Kupffer cells (KCs), which are resident macrophages in the liver, constitute the largest population of resident tissue macrophages in the body.^[^ ^4^ ^]^ Under physiological conditions, small amounts of LPS are eliminated primarily by KCs, resulting in immune tolerance in the liver.^[^ ^5^ ^]^ While during endotoxemia‐induced liver injury, excess LPS binds to macrophage Toll‐like receptor 4 (TLR4), promoting the phosphorylation of inhibitor of κB kinase (IKK).^[^ ^6^ ^]^ This leads to the phosphorylation and subsequent degradation of inhibitor of κB (IκB), which in turn triggers the nuclear translocation of the nuclear factor κB (NFκB) complex.^[^ ^7^, ^8^ ^]^ Macrophages can be polarized to a classic M1 phenotype characterized by the expression of inducible nitric oxide synthase (iNOS) and cluster of differentiation 86 (CD86).^[^ ^9^ ^]^ Hyperinflammation caused by macrophages results in hepatocyte damage and lethal liver injury,^[^ ^8^ ^]^ and the available evidence suggests that TLR4 plays a role in the pathogenesis of endotoxin‐induced liver injury.^[^ ^7^ ^]^ Thus, a better understanding of the molecular mechanisms involved in macrophage function may facilitate the development of new therapeutic strategies for liver injury.

Constitutive androstane receptor (CAR), a nuclear receptor and xenobiotic sensor, is distributed mainly in the liver and gastrointestinal tract.^[^ ^10^ ^]^ In the context of hepatic xenobiotic metabolism, CAR regulates the expression of proteins involved in detoxification and clearance, such as UDP glucuronosyltransferase family 1 member A1 (UGT1A1), cytochrome P450 family 2 subfamily B polypeptide 10 (CYP2B10), and sulfotransferase family 2A member 1 (SULT2A1).^[^ ^11^ ^]^ Furthermore, CAR has been shown to modulate many biological functions, including the maintenance of energy homeostasis, cellular proliferation, and cancer development.^[^ ^12^, ^13^, ^14^, ^15^ ^]^ Recently, evidence has shown that CAR is involved in inflammation‐related diseases. The activation of CAR inhibits leukocyte adhesion to dysfunctional endothelial cells.^[^ ^16^ ^]^ Moreover, CAR activation benefits inflammatory bowel disease by reducing damage and promoting intestinal mucosal repair.^[^ ^17^ ^]^ Nevertheless, whether and how CAR participates in the pathological process of endotoxin‐induced liver injury remains largely unknown.

The present study aimed to investigate the modulatory effect of CAR on hepatic macrophages and elucidate its pathophysiological role in endotoxemic liver injury. We report that CAR is abundantly expressed in human and murine macrophages and that activating CAR reduces the inflammatory response in macrophages. Functionally, CAR activation suppressed the release of proinflammatory cytokines by macrophages, attenuated hepatocyte death, and ameliorated LPS/D‐galactosamine (GaIn)‐induced liver injury. Mechanistic studies revealed that CAR was essential in macrophages and interacted with *Tlr4* to exert a protective effect on liver injury. These findings reveal the function of CAR in macrophages and its beneficial role in endotoxin‐induced liver injury, providing a promising therapeutic strategy based on the use of CAR agonists for inflammatory liver diseases.

## 2 Results

### 2.1 CAR is Expressed in Macrophages and Attenuates Hyperinflammatory Polarization

**Figure 1:**
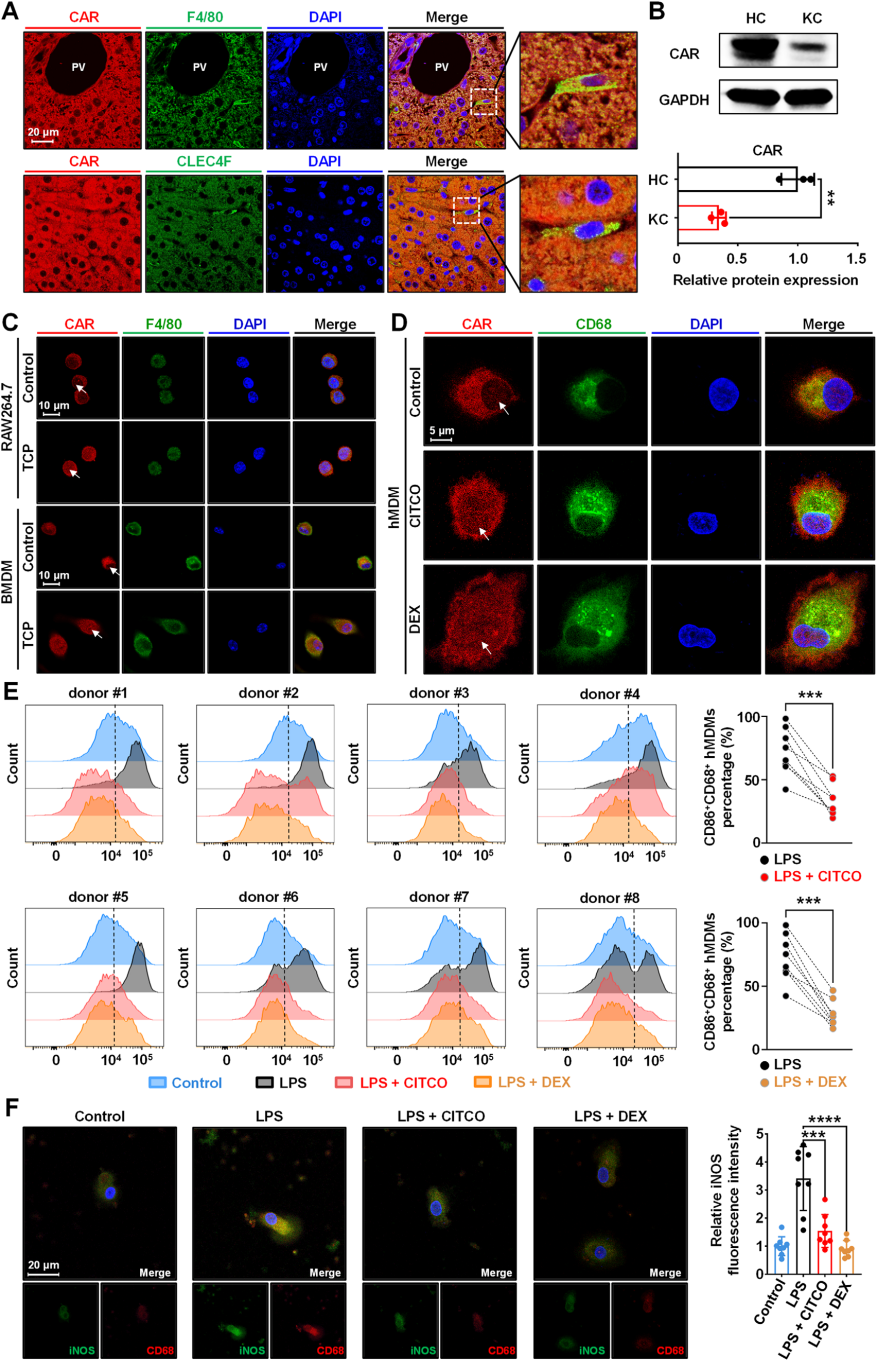
Expression and modulation of CAR in macrophages. A) Localization and expression of CAR in murine liver, depicting CAR, F4/80 (mouse macrophage marker) or CLEC4F (KC marker), and DAPI (nucleus) nuclear staining. B) Immunoblotting of HC and KC lysates for CAR and GAPDH. C) Immunofluorescence images of the nuclear translocation of CAR (red) in RAW264.7 and BMDM cells after TCP incubation. D) Immunofluorescence images of the nuclear translocation of CAR in hMDMs after CITCO or DEX incubation (CD68, macrophage marker, green; CAR, red). E) Flow cytometric histogram and proportion of CD86^+^CD68^+^ hMDMs. hMDMs were incubated with vehicle or LPS (1 µg mL^−1^ for 24 h) with or without CITCO or DEX (1 µM for 48 h) after isolation and differentiation from healthy donors (*n* = 8, four males and four females). F) Immunofluorescence staining and quantification of iNOS fluorescence intensity in hMDMs. Data are means ± SD. ^**^ *p *< 0.01, ^***^ *p *< 0.001, and ^****^ *p *< 0.0001. Statistical analyses were performed using unpaired or paired Student's *t*‐test, or one‐way ANOVA with Dunnett's multiple comparisons test.

To demonstrate the CAR profile on macrophages, we first examined the localization and expression of CAR in mouse and human macrophages. Immunofluorescence staining revealed that cells positive for F4/80 (a marker for macrophages) and C‐type lectin domain family 4 member F (CLEC4F, a marker for KCs) both expressed CAR (**Figure** **1**A). Moreover, the abundance of CAR in KCs and hepatocytes (HCs) was investigated after the cells were isolated from the mouse liver. The results revealed that the protein expression level of CAR in KCs was ≈34% of that in HCs (Figure 1B). To assess whether macrophage CAR can be activated by agonists, murine bone marrow‐derived macrophages (BMDMs) and RAW264.7 cells were treated with 1,4‐bis[2‐(3,5‐dichloropyridyloxy)]benzene (TCP). Immunofluorescence analysis revealed that CAR was highly expressed in the cytoplasm of macrophages and underwent nuclear translocation upon stimulation with TCP (Figure 1C).

To extend these observations from mice to humans, human monocyte‐derived macrophages (hMDMs) were isolated and differentiated from eight healthy donors. The hMDMs were treated with 6‐(4‐chlorophenyl)imidazo[2,1‐b][1,3]thiazole‐5‐carbaldehyde‐O‐(3,4‐dichlorobenzyl)oxime (CITCO, a human‐specific CAR agonist) and dexamethasone (DEX, a classical CAR agonist used in the clinic) to activate CAR. As shown in Figure 1D, nuclear translocation of CAR also occurred in the CITCO‐ and DEX‐treated groups, suggesting that CAR could be activated by exogenous stimulants in hMDMs. We further investigated the effect of CAR activation in LPS‐induced inflammatory M1 macrophages. Flow cytometry analysis revealed that the percentage of CD86^+^ (M1 marker) cells in the CITCO and DEX treatment groups was markedly lower than that in the LPS group (Figure 1E). Furthermore, iNOS is a pivotal downstream mediator of inflammation and a marker of M1 macrophages. The fluorescence intensity of iNOS was significantly increased in LPS‐treated macrophages, whereas CITCO and DEX treatment reduced the expression of iNOS in hMDMs (Figure 1F). These results suggest that CAR is functional in human and murine macrophages and that macrophage CAR activation may modulate immune responses.

### 2.2 Activation of CAR Alleviates Inflammation and Protects Against Endotoxin‐Induced Liver Injury

**Figure 2:**
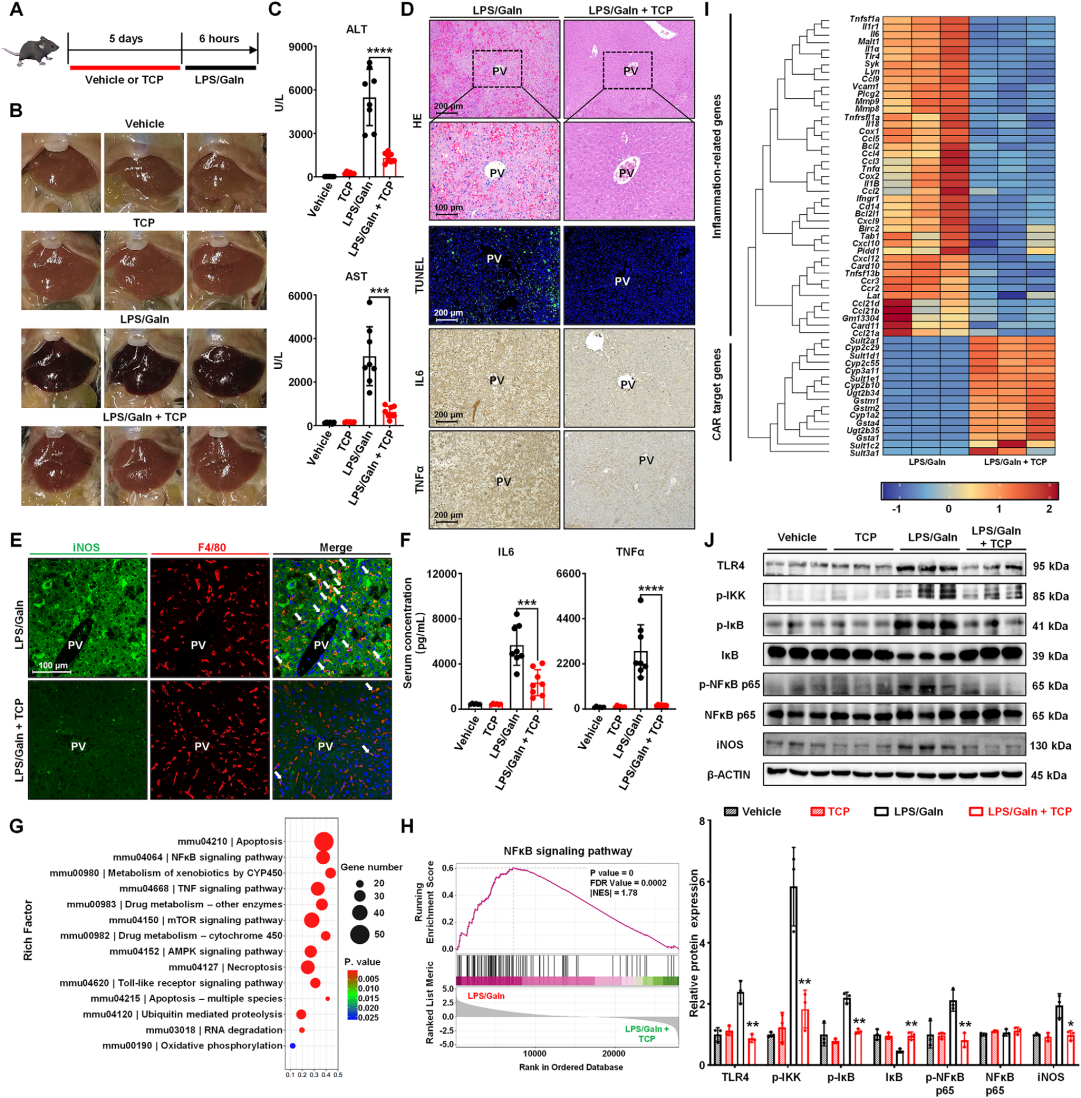
CAR activation alleviates inflammation and protects against endotoxin‐induced liver injury. A) Mice were treated with TCP (3 mg kg^−1^ d^−1^) or vehicle (corn oil) for 5 days, LPS/GaIn (100 µg kg^−1^ and 700 mg kg^−1^, respectively) was administered for 6 h (*n* = 6 for vehicle and TCP group, *n* = 8 for LPS/GaIn and LPS/GaIn + TCP group). B) Representative gross pictures of the liver. C) Serum ALT and AST levels. D) Representative H&E, TUNEL, IL6, and TNFα staining of liver sections. E) Immunofluorescence staining of iNOS (green) and F4/80 (red). iNOS^+^F4/80^+^ macrophages indicated with white arrows. F) ELISA assay of serum IL6 and TNFα levels. G) GO enrich analysis between LPS/GaIn and LPS/GaIn + TCP group (*n* = 3). H) Different genes between the LPS/GaIn and LPS/GaIn + TCP group enriched in NFκB signaling pathway via GSEA (*p* value = 0.0000, FDR value = 0.0002, |NES| = 1.78). I) Heatmap of inflammation‐related and CAR‐targeted differentially expressed genes after TCP treatment in LPS challenge. J) The protein expression of TLR4, p‐IKK, p‐IκB, IκB, p‐NFκB p65, NFκB p65, and iNOS in mouse liver. Data are means ± SD. ^*^ *p* < 0.05, ^**^ *p* < 0.01, ^***^ *p* < 0.001, and ^****^ *p* < 0.0001. Statistical analyses were performed using an unpaired Student's *t*‐test.

To investigate whether CAR activation could rescue endotoxin‐induced liver injury, liver pathological characteristics and inflammatory infiltration following LPS/GaIn challenge were investigated (**Figure** **2**A). Western blotting result showed that LPS/GaIn significantly decreased CAR expression, while TCP increased the nuclear and total CAR protein levels (Figure S1G, Supporting Information). Additionally, the expression of proteins downstream of CAR (CYP2B10, UGT1A1, and SULT2A1) was increased after TCP treatment, indicating successful activation of hepatic CAR (Figure S1H, Supporting Information). In the context of LPS exposure, TCP treatment significantly alleviated the severe liver congestion induced by LPS and decreased the serum levels of alanine transaminase (ALT, −77%, *p *< 0.0001) and aspartate transaminase (AST, −82%, *p *< 0.0001) (Figure 2B,C). To further evaluate the pathological changes induced by LPS/GaIn, we performed hematoxylin and eosin (H&E) and terminal deoxynucleotidyl transferase dUTP nick end labeling (TUNEL) staining of mouse liver sections. As expected, TCP treatment considerably attenuated the multifocal hemorrhage and extensive necrosis around the hepatic portal vein (PV) caused by LPS/GaIn (Figure 2D; Figure S1A, Supporting Information). The number of TUNEL^+^ cells was also significantly reduced, suggesting that TCP treatment reduced hepatocyte death (Figure 2D; Figure S1B, Supporting Information). Most strikingly, in our evaluation of hepatic inflammation, CAR activation significantly reduced the number of iNOS^+^F4/80^+^ macrophages (Figure 2E; Figure S1C, Supporting Information) and decreased the infiltration of IL6 and TNFα in the liver compared with those in the LPS/GaIn group (Figure 2D; Figure S1D,E, Supporting Information). Furthermore, the enzyme‐linked immunosorbent assay (ELISA) results revealed a reduction in the IL6 and TNFα levels in the serum (Figure 2F). These results suggest that CAR activation attenuated liver inflammation and endotoxemic liver injury.

To identify candidate genes that may be associated with CAR activation during liver injury, RNA sequencing (RNA‐seq) was performed. Gene Ontology (GO) analysis revealed that the genes were differentially expressed between the LPS/GaIn + TCP group and the LPS/GaIn group were highly enriched in the apoptosis, TLR4/NFκB and CYP450 pathways (Figure 2G). Gene set enrichment analysis (GSEA) revealed that the NFκB signaling pathway was downregulated in the LPS/GaIn + TCP group compared with the LPS/GaIn group (*P* = 0.0000, FDR value = 0.0002, |NES| = 1.78; Figure 2H). We further validated the proteins related to the TLR4/NFκB signaling pathway. CAR activation significantly downregulated the expression of TLR4, p‐IKK, p‐IκB, p‐NFκB p65, and iNOS and upregulated the expression of IκB (Figure 2J; Figure S1I, Supporting Information). The efficacy of CAR activation on the expression of inflammation‐related genes and proinflammatory cytokines such as *Il6, Il1β, Tnfα*, C C motif ligand 2 (*Ccl2*), *Ccl3*, and cyclooxygenase 2 (*Cox2*) was verified, and the results were consistent with the RNA‐seq results (Figure 2I; Figure S1F, Supporting Information). Taken together, these results suggest that CAR activation ameliorates LPS/GaIn‐induced liver injury by suppressing the inflammatory response and that this effect may be exerted via regulation of the TLR4/NFκB signaling pathway.

### 2.3 CAR Activation Reduces Proinflammatory Macrophages and Attenuates Inflammation via Macrophage‐Hepatocyte Crosstalk

**Figure 3:**
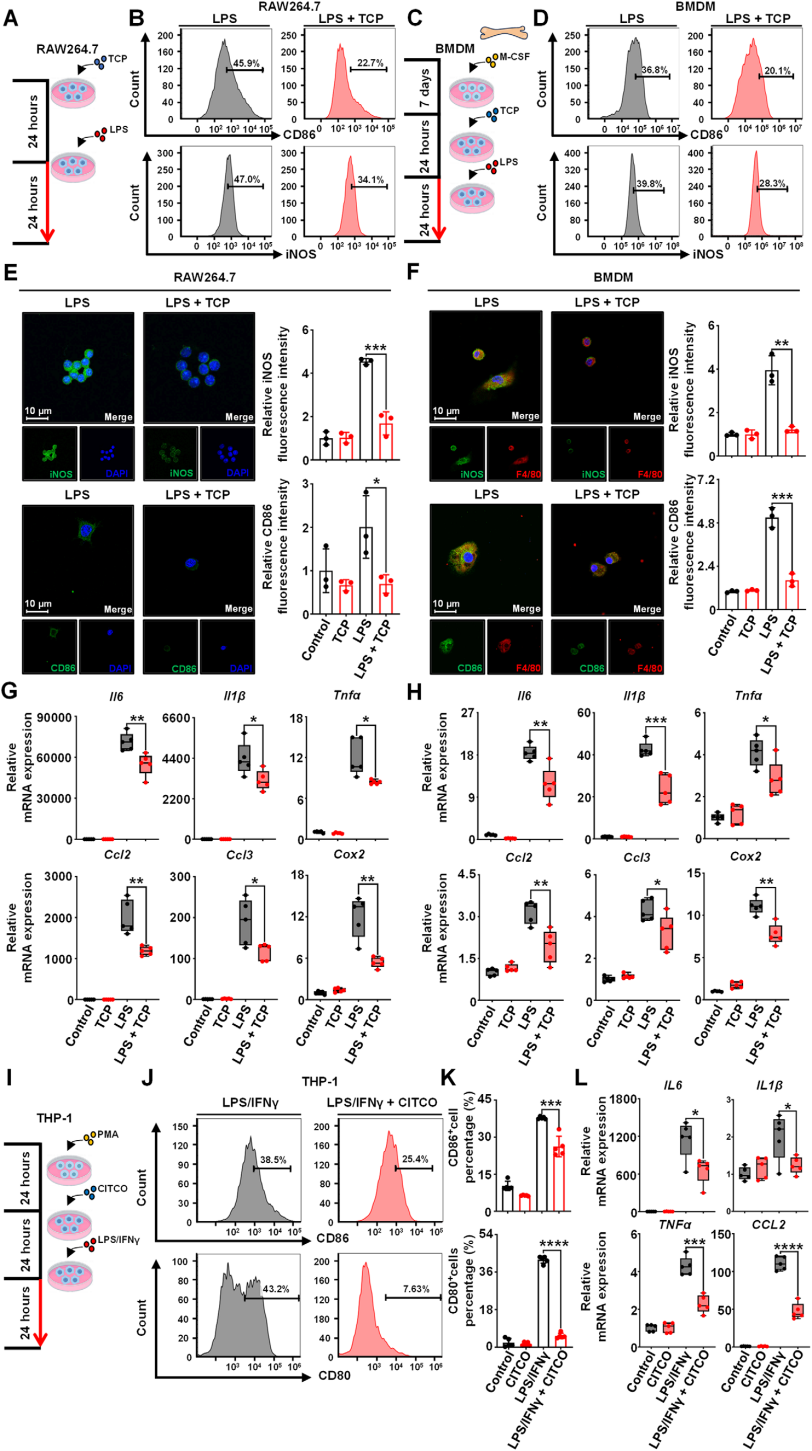
The effects of CAR activation on RAW264.7, BMDM, and THP‐1 macrophages. A, C) RAW264.7 cells (A) or BMDMs (C) were incubated with vehicle or LPS (1 µg mL^−1^, for 24 h) with or without TCP (5 µM, for 48 h) (*n *= 5). B, D) Flow cytometric histogram of CD86^+^ or iNOS^+^ cells in RAW264.7 cells (B) or BMDMs (D) after TCP treatment. E, F) Immunofluorescence staining and quantification of CD86 and iNOS fluorescence intensity in RAW264.7 cells (E) or BMDMs (F). G, H) qRT‐PCR comparing relative levels of the inflammatory‐related genes (*Il6, Il1β, Tnfα, Ccl2*, *Ccl3*, and *Cox2*) in RAW264.7 cells (G) or BMDMs (H). I) THP‐1 macrophages were incubated with vehicle or LPS/IFNγ (100 ng mL^−1^ and 20 ng mL^−1^, for 24 h) with or without CITCO (1 µM, for 48 h) (*n *= 5). J, K) Flow cytometric histogram and proportion of CD86^+^ or CD80^+^ cells in THP‐1 macrophages incubated with CITCO. L) The expression of the inflammatory‐related genes (*IL6, IL1β, TNFα*, and *CCL2*). Data are means ± SD. ^*^ *p* < 0.05, ^**^ *p *< 0.01, ^***^ *p *< 0.001, and ^****^ *p *< 0.0001. Statistical analyses were performed using an unpaired Student's *t*‐test.

Since the inflammatory effects of LPS on the liver are initiated primarily by macrophages, we next examined the modulatory effects of CAR activation on macrophage phenotype and function in vitro. Murine macrophage RAW264.7 cells and BMDMs were pretreated with TCP and then incubated with LPS (**Figure** **3**A,C). The expression of CYP2B10, UGT1A1, and SULT2A1 was increased after TCP treatment, suggesting successful activation of CAR in macrophages (Figure S2C, Supporting Information). The results showed that TCP treatment reduced the proportions of CD86^+^ and iNOS^+^ cells (Figure 3B,D; Figure S2A,B,E,F, Supporting Information) and decreased the fluorescence intensity of CD86 and iNOS in RAW264.7 cells and BMDMs (Figure 3E,F). Moreover, TCP treatment significantly reduced the expression of inflammatory genes (*Il6, Il1β, Tnfα, Ccl2*, *Ccl3*, and *Cox2*) in RAW264.7 cells and BMDMs (Figure 3G,H), which was consistent with the in vivo results.

Human THP‐1 macrophages were incubated with the specific CAR agonist CITCO and then treated with LPS/Interferon‐gamma (IFNγ) (Figure 3I). THP‐1 macrophages were successfully activated by CITCO (Figure S2G, Supporting Information). Flow cytometry revealed that the proportions of CD86^+^ and CD80^+^ (human M1 marker) cells in the LPS/IFNγ + CITCO group were significantly lower than those in the LPS/IFNγ group (Figure 3J,K). Additionally, the levels of the inflammation‐related genes *IL6, IL1β, TNFα*, and *CCL2* in THP‐1 macrophages were significantly decreased by CITCO treatment (Figure 3L).

**Figure 4:**
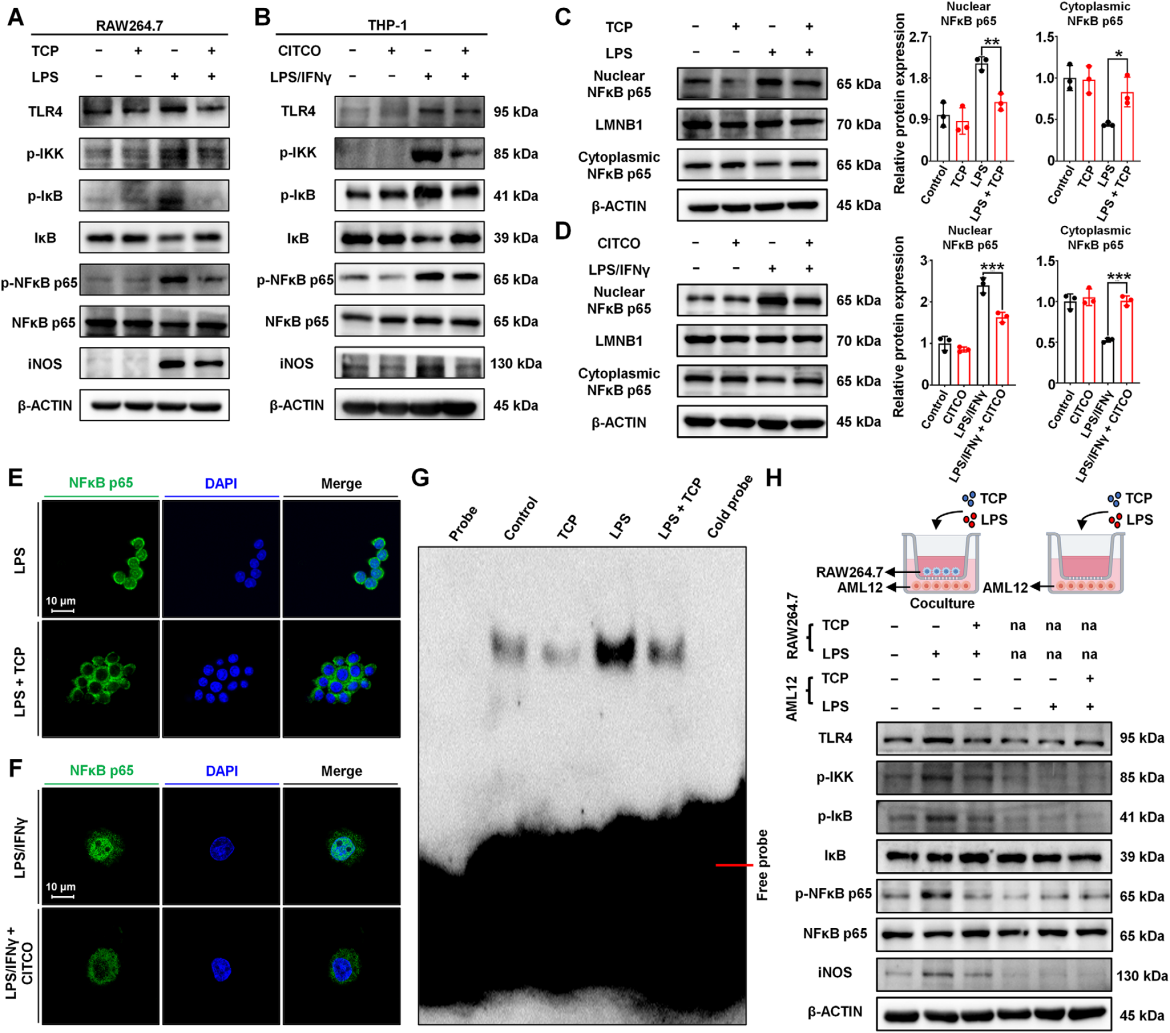
CAR activation regulates the TLR4/NFκB pathway in RAW264.7 and THP‐1 cells. A, B) The protein expression of total TLR4, p‐IKK, p‐IκB, IκB, p‐NFκB p65, NFκB p65, and iNOS in RAW264.7 cells (A) and THP‐1 macrophages (B). C, D) The protein expression of nuclear NFκB p65 and cytoplasmic NFκB p65 in RAW264.7 (C) and THP‐1 macrophages (D) (*n *= 3). E, F) Immunofluorescence staining of nuclear NFκB p65 in RAW264.7 (E) and THP‐1 macrophages (F). G) EMSA assay of NFκB binding to DNA in RAW264.7 cells. H) Coculture system of RAW264.7 and AML12 cells. RAW264.7 cells were incubated with vehicle or LPS (1 µg mL^−1^, for 24 h) with or without TCP (5 µM, for 48 h), and then cocultured with AML12 for 24 h in the coculture group; AML12 cells alone were incubated with vehicle or LPS (1 µg mL^−1^, for 24 h) with or without TCP (5 µM, for 48 h), na: not applicable. The protein expression of TLR4, p‐IKK, p‐IκB, IκB, p‐NFκB p65, NFκB p65, and iNOS in AML12 cells. Data are means ± SD. ^*^ *p *< 0.05, ^**^ *p *< 0.01, and ^***^ *p *< 0.001. Statistical analyses were performed using an unpaired Student's *t*‐test.

To confirm the above results, the TLR4/NFκB signaling pathway in RAW264.7 and THP‐1 macrophages was investigated. CAR activation significantly decreased the expression of TLR4, p‐IKK, p‐IκB, p‐NFκB p65, and iNOS and increased the expression of IκB in both RAW264.7 and THP‐1 macrophages (**Figure** **4**A,B; Figure S2D,H, Supporting Information). LPS binds to macrophage TLR4 and subsequently induces the nuclear translocation of NFκB, and the binding of the NFκB subunit to DNA initiates the transcription of downstream inflammatory genes. The effects of CAR activation on NFκB nuclear translocation were explored. Immunofluorescence staining revealed that LPS stimulation increased NFκB p65 nuclear translocation, and this effect was reversed by treatment with TCP in RAW264.7 cells or CITCO in THP‐1 macrophages, respectively (Figure 4C–F). Moreover, electrophoretic mobility shift assay (EMSA) results confirmed that CAR activation resulted in decreased DNA‐binding activity of NFκB in macrophages (Figure 4G).

To study the effect of the CAR activation‐modulated immunological cascade in macrophages on hepatocytes, RAW264.7 cells (macrophages) were cocultured with AML12 cells (hepatocytes). After a coculture with LPS‐stimulated RAW264.7 cells, the expression of immune‐related proteins (TLR4, p‐IKK, p‐IκB, p‐NFκB p65, and iNOS) in the AML12 cells in the lower wells was increased, whereas that of IκB was decreased, and these effects were reversed by TCP treatment of the RAW264.7 cells in the upper well (Figure 4H; Figure S2I, Supporting Information). These findings revealed that CAR activation suppressed proinflammatory macrophage activation and crosstalk with hepatocytes.

### 2.4 Loss of CAR in Macrophages Inhibits the Hepatoprotective Effect of CAR Activation During Endotoxin‐Induced Liver Injury

**Figure 5:**
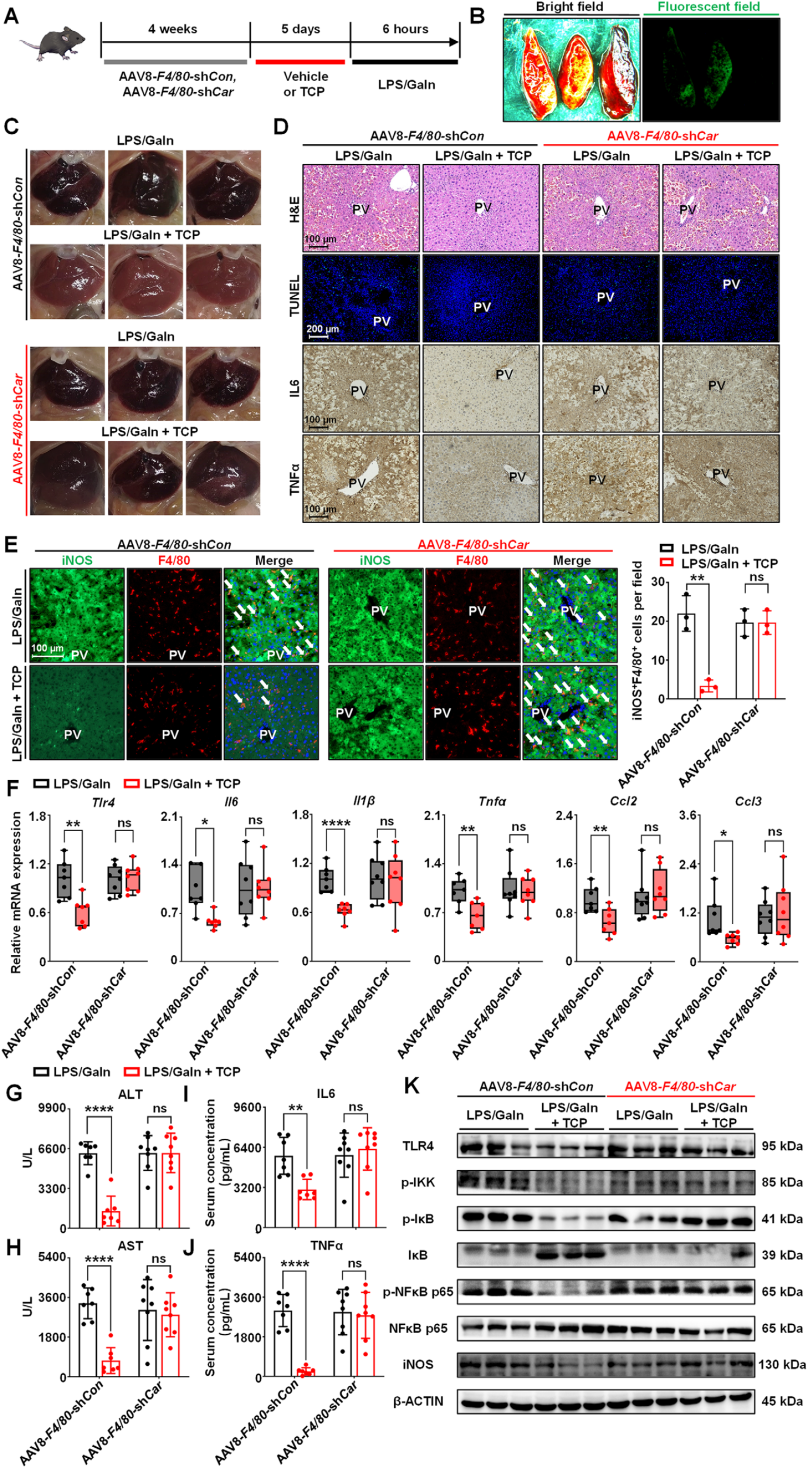
Ablation of CAR in macrophages impairs the hepatoprotective effect of CAR activation in LPS/GaIn‐induced liver injury. A) Mice were intravenously injected with AAV8‐*F4/80*‐sh*Con* or AAV8‐*F4/80*‐sh*Car* for four weeks to generate macrophage‐specific *Car* deficiency models. TCP (3 mg kg^−1^ d^−1^) was intraperitoneally injected for 5 days, and LPS/GaIn (100 µg kg^−1^ and 700 mg kg^−1^, respectively) was injected 2 h after the last injection, and the mice were sacrificed 6 h later (*n* = 7 for AAV8‐*F4/80*‐sh*Con* group, and *n *= 8 for AAV8‐*F4/80*‐sh*Car* group). B) Representative fluorescent images of livers treated with AAV8‐*F4/80*‐sh*Con* (left tissue), AAV8‐*F4/80*‐sh*Car* (middle tissue), and saline (right tissue). C) Representative gross pictures of the liver. D) Representative H&E, TUNEL, IL6, and TNFα staining of liver sections. E) Immunofluorescence staining and quantification of iNOS (green) and F4/80 (red). White arrows indicated the iNOS^+^F4/80^+^ macrophages. F) The gene expression of the *Tlr4*, *Il6*, *Il1β*, *Tnfα*, *Ccl2*, and *Ccl3*. G, H) Serum ALT (G) and AST (H) levels. I, J) ELISA assay of serum IL6 (I) and TNFα (J) levels. K) The protein expression of TLR4, p‐IKK, p‐IκB, IκB, p‐NFκB p65, NFκB p65, and iNOS in the liver. Data are means ± SD. ^*^ *p *< 0.05, ^**^ *p *< 0.01, and ^****^ *p *< 0.0001. ns: not significant. Statistical analyses were performed using an unpaired Student's *t*‐test.

To assess the role of macrophage CAR in endotoxin‐induced liver injury, we generated macrophage‐specific *Car* knockdown mice and negative control mice by using adeno‐associated virus 8 (AAV8)‐*F4/80*‐*Car*‐shRNA‐Zsgreen (AAV8‐*F4/80*‐sh*Car*) and AAV8‐*F4/80*‐shRNA‐Zsgreen (AAV8‐*F4/80*‐sh*Con*), respectively (**Figure** **5**A). Successful transfection and deletion of the macrophage CAR were confirmed by liver fluorescence imaging, immunofluorescence, and western blotting (Figure 5B; Figure S3A,B, Supporting Information). Knockdown of CAR in macrophages did not influence the activation of CAR by TCP in the whole liver, as evidenced by the expression of proteins downstream of CAR (Figure S3G, Supporting Information). In the AAV8‐*F4/80*‐sh*Con* group, the administration of TCP still exerted a protective effect against LPS/GaIn‐induced liver injury. However, the administration of TCP in the AAV8‐*F4/80*‐sh*Car* group failed to alleviate hepatic congestion and necrosis (Figure 5C,D; Figure S3C,D, Supporting Information) or decrease the serum levels of ALT and AST (Figure 5G,H). These results suggest that the absence of CAR in macrophages compromises the protective effect of CAR activation on endotoxemic liver injury.

The immunomodulatory effects of CAR were further investigated. In AAV8‐*F4/80*‐sh*Con* mice, TCP treatment alleviated the production of IL6 and TNFα in the liver and serum (Figure 5D,I,J; Figure S3E,F, Supporting Information), reduced the number of iNOS^+^F4/80^+^ (M1) macrophages induced by LPS/GaIn (Figure 5E) and decreased the expression of the inflammation‐related genes *Tlr4, Il6, Il1β, Tnfα, Ccl2*, and *Ccl3* (Figure 5F). Additionally, after CAR knockdown in liver macrophages, we observed no significant differences between vehicle‐ and TCP‐treated endotoxemic mice (Figure 5D–J; Figure S3C–F, Supporting Information). Next, the modulation of the TLR4/NFκB signaling pathway was analyzed. The results revealed that CAR activation significantly reduced the expression of TLR4, p‐IKK, p‐IκB, p‐NFκB p65, and iNOS and increased the expression of IκB in the AAV8‐*F4/80*‐sh*Con* group, and this effect was not observed in the mice injected with AAV8‐*F4/80*‐sh*Car* (Figure 5K; Figure S3H, Supporting Information). Taken together, these findings revealed that the macrophage CAR is essential for CAR activation‐induced protection against endotoxemic liver injury.

### 2.5 CAR Activation Protects against LPS/Gain‐Induced Liver Injury in a TLR4‐Dependent Manner

**Figure 6:**
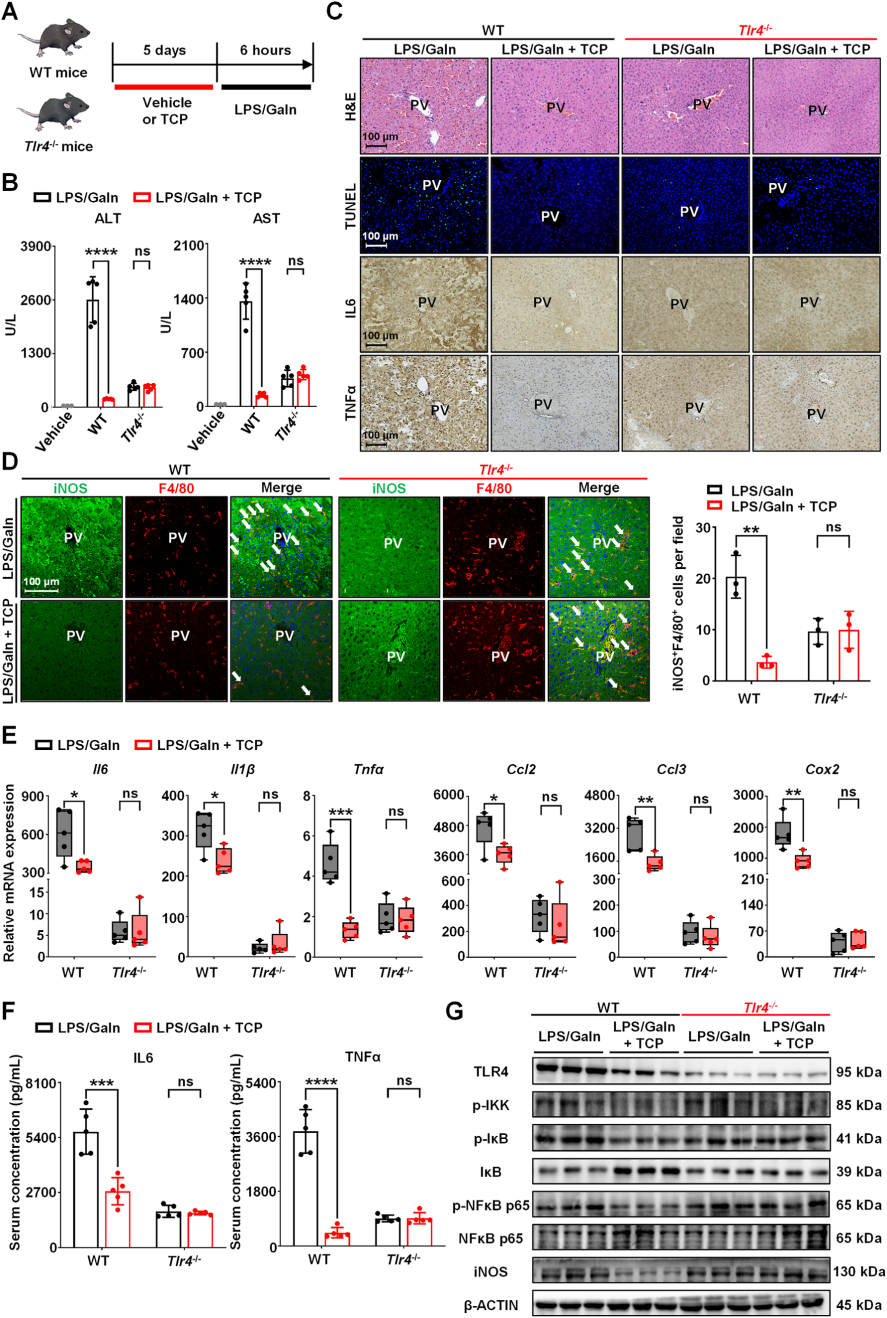
CAR activation protects against endotoxin‐induced liver injury in a TLR4‐dependent manner. A) WT or *Tlr4* ^−/−^ mice were treated with TCP (3 mg kg^−1^ d^−1^) or vehicle (corn oil) for 5 days, then LPS/GaIn (100 µg kg^−1^ and 700 mg kg^−1^, respectively) was administered, and all mice were sacrificed 6 h post‐injection (*n* = 5). B) Serum ALT and AST levels. C) Representative H&E, TUNEL, IL6, and TNFα staining of liver sections. D) Immunofluorescence staining and quantification of iNOS (green) and F4/80 (red). E) The expression of the inflammatory‐related genes (*Il6*, *Il1β*, *Tnfα*, *Ccl2*, *Ccl3*, and *Cox2*) in the liver. F) ELISA assay of serum IL6 and TNFα levels. G) The protein expression of TLR4, p‐IKK, p‐IκB, IκB, p‐NFκB p65, NFκB p65, and iNOS in the liver. Data are means ± SD. ^*^ *p *< 0.05, ^**^ *p *< 0.01, ^***^ *p *< 0.001, and ^****^ *p *< 0.0001. ns: not significant. Statistical analyses were performed using an unpaired Student's *t*‐test.

Given that the role of TLR4 in endotoxin‐induced liver injury is well documented, we performed further studies to assess the contribution of TLR4 to CAR‐attenuated liver injury. *Tlr4*‐knockout (*Tlr4* ^−/−^) and wild‐type (WT) mice were treated with TCP or vehicle and subjected to LPS/GaIn challenge as shown in **Figure** **6**A. A quantitative real‐time polymerase chain reaction (qRT‐PCR) assay revealed a successful knockout of TLR4 in the liver (Figure S4A, Supporting Information). Immunoblotting data revealed that after TCP treatment, CAR was still activated in the WT and *Tlr4* ^−/−^ mice (Figure S4B, Supporting Information). These results suggested that *Tlr4* knockout could partially alleviate LPS/GaIn‐induced liver injury; in parallel, we found that TCP treatment failed to attenuate liver dysfunction or hepatocyte death in *Tlr4* ^−/−^ mice, as evidenced by the high serum ALT and AST levels caused by inflammation (Figure 6B), as well as the presence of liver damage and unreduced TUNEL^+^ cells (Figure 6C; Figure S4C,D, Supporting Information). Consistent with these findings, CAR activation failed to inhibit proinflammatory activation and cytokine production in *Tlr4* ^−/−^ mice (Figure 6C–F; Figure S4E,F, Supporting Information). A more in‐depth analysis revealed that CAR activation reduced the LPS/GaIn‐induced increase in TLR4, p‐IKK, p‐IκB, p‐NFκB p65, and iNOS expression and prevented IκB degradation in WT mice. In contrast, the administration of TCP failed to reverse the observed protein expression changes in *Tlr4* ^−/−^ mice (Figure 6G; Figure S4G, Supporting Information). These results suggest that TLR4 plays an important role in the hepatoprotective effect of CAR during LPS/GaIn‐induced liver injury.

### 2.6 CAR Binds to *Tlr4* and Modulates the TLR4 Signaling Pathway

**Figure 7:**
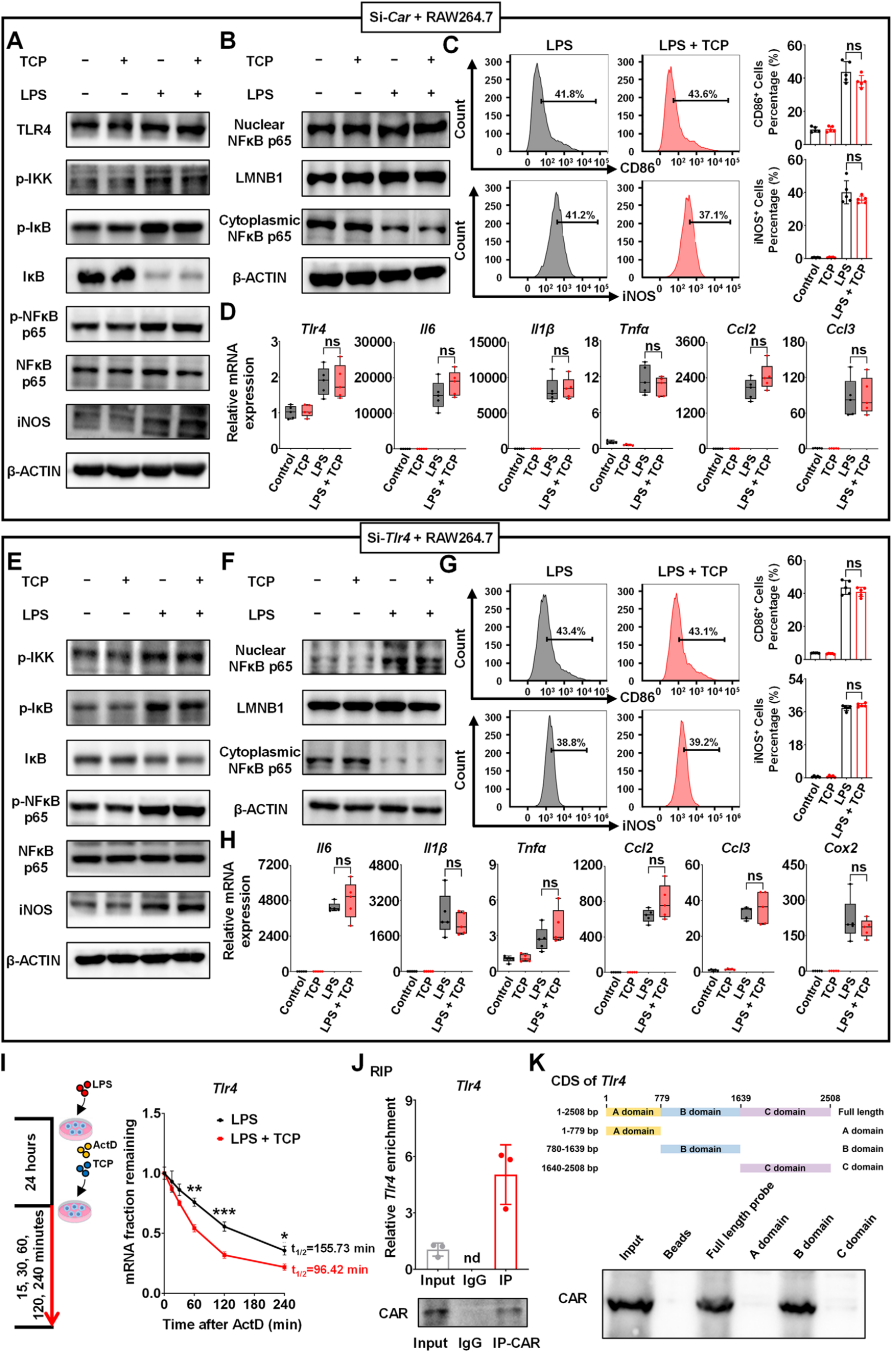
CAR binds to *Tlr4* and modulates the TLR4 signaling pathway. A–D) RAW264.7 cells were transfected with siRNA for 24 h to knock down *Car*, and then incubated with vehicle or LPS (1 µg mL^−1^, for 24 h) with or without TCP (5 µM, for 48 h) (*n *= 5). A) The expression of proteins in the TLR4/NFκB pathway. B) The protein expression of nuclear and cytoplasmic NFκB p65. C) Flow cytometric histogram and proportion of CD86^+^ and iNOS^+^ cells. D) Analysis of the inflammation‐related genes. E‐H) RAW264.7 cells were transfected with siRNA for 24 h to knock down *Tlr4*, and then incubated with vehicle or LPS (1 µg mL^−1^, for 24 h) with or without TCP (5 µM, for 48 h) (*n *= 5). E) The expression of proteins in TLR4/NFκB pathway. F) The protein expression of nuclear and cytoplasmic NFκB p65. G) Flow cytometric histogram and proportion of CD86^+^ and iNOS^+^ cells. H) Analysis of the inflammation‐related genes. I) RAW264.7 cells were treated with LPS (1 µg mL^−1^, for 24 h), and further treated with 20 µg mL^−1^ ActD chase with or without 5 µM TCP. mRNA levels of *Tlr4* were measured at different time points. J) RNA immunoprecipitation of CAR‐bound *Tlr4*. Nd: not determined. K) RNA pull‐down experiments were performed with CAR and biotinylated *Tlr4*. Data are means ± SD. ^*^ *p *< 0.05, ^**^ *p *< 0.01, and ^***^ *p *< 0.001. ns: not significant. Statistical analyses were performed using an unpaired Student's *t*‐test.

To assess whether CAR‐mediated LPS‐induced inflammation was dependent on TLR4 in vitro, small interfering RNAs (siRNAs) were used to knock down *Car* (Figure S5A, Supporting Information) and *Tlr4* (Figure S5D, Supporting Information). After *Car* silencing, TCP treatment completely abolished the downregulation of TLR4, p‐IKK, p‐IκB, p‐NFκB p65, and iNOS expression and the upregulation of IκB expression (**Figure** **7**A; Figure S5C, Supporting Information). Moreover, after the *Car* knockdown, TCP treatment failed to modulate NFκB p65 nuclear translocation;, the proportions of M1 macrophages positive for CD86 and iNOS, and the expression of the inflammation‐related genes *Tlr4*, *Il6, Il1β, Tnfα, Ccl2*, and *Ccl3* (Figure 7B–D; Figure S5B, Supporting Information). Similar results were obtained after si‐*Tlr4* treatment, as the effects of CAR activation on genes related to inflammation (*Il6, Il1β, Tnfα, Ccl2*, *Ccl3*, and *Cox2*), M1 macrophages (CD86^+^ and iNOS^+^), and TLR4 pathway regulation (p‐IKK, p‐IκB, IκB, p‐NFκB p65, and iNOS) disappeared (Figure 7E–H; Figure S5E,F, Supporting Information).

The activation of CAR downregulated *Tlr4* expression in vivo and in vitro (Figure S5G,H, Supporting Information). Thus, we speculated that CAR may act post‐transcriptionally to attenuate the expression of *Tlr4*. An actinomycin D (ActD) chase experiment was conducted; RAW264.7 cells were treated with 1 µg mL^−1^ LPS for 24 h and then cultured with ActD and TCP (Figure 7I). The *Tlr4* levels indicated that TCP decreased the stability of *Tlr4* (the t_1/2_ of *Tlr4* in the LPS group was 155.7 min, whereas that in the LPS + TCP group was 96.4 min, Figure 7I). Moreover, RNA‐binding protein immunoprecipitation (RIP) assays revealed that *Tlr4* could be immunoprecipitated by CAR (Figure 7J; Figure S5I, Supporting Information). We next conducted a biotin‐labeled RNA pull‐down experiment, and western blotting analysis of the pulled‐down products confirmed that CAR bound to the B domain (780–1639 bp) of the CDS of *Tlr4* (Figure 7K). These findings strongly suggest that CAR interacts with *Tlr4* and reduces the stability of *Tlr4*.

### 2.7 Clinical Drugs that Activate CAR Alleviate LPS/Gain‐Induced Liver Injury, and CAR Activation Promotes Liver Recovery in the Context of Infection after Liver Surgery

**Figure 8:**
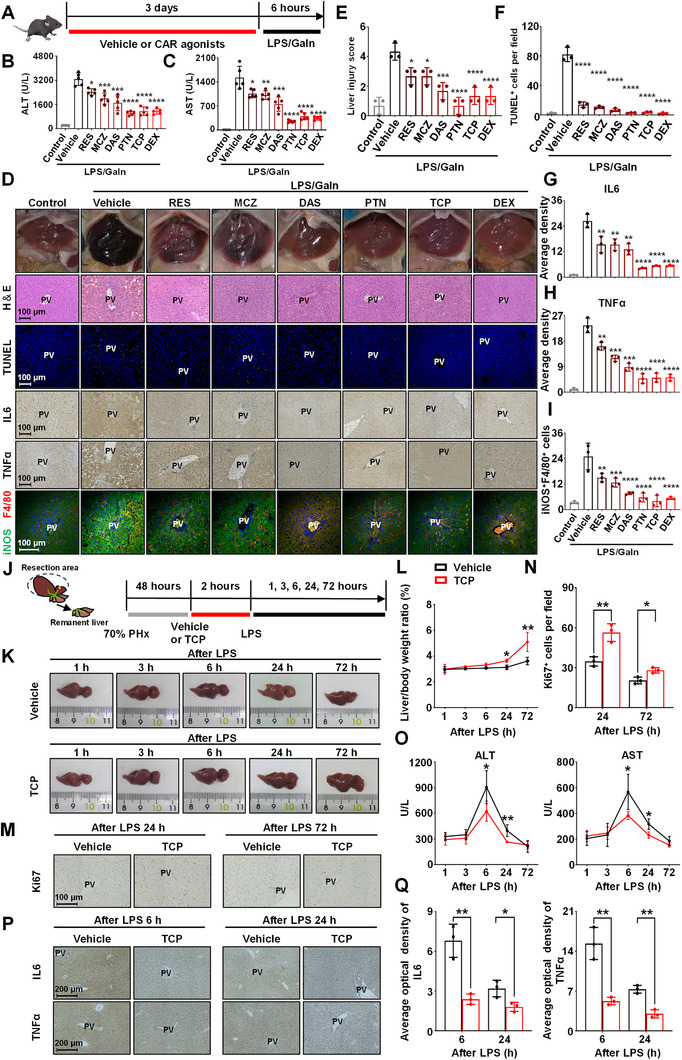
Effects of CAR agonists on endotoxin‐induced liver injury and liver surgical infection. A) Mice were treated with 3 mg kg^−1^ d^−1^ TCP, 50 mg kg^−1^ d^−1^ DEX, 50 mg kg^−1^ d^−1^ PTN, 100 mg kg^−1^ d^−1^ RES, 100 mg kg^−1^ d^−1^ MCZ, 100 mg kg^−1^ d^−1^ DAS, or vehicle (corn oil) for 3 days prior to LPS/GaIn exposure (100 µg kg^−1^ and 700 mg kg^−1^, respectively), and mice were sacrificed 6 h after last injection (*n* = 5). B, C) Serum ALT (B) and AST (C) levels. D) Representative gross pictures of the liver, H&E, TUNEL, IL6, and TNFα staining, F4/80 (red) and iNOS (green) double staining of liver sections. E) Histological score of the H&E. F) Quantification of TUNEL^+^ cells. G, H) Quantification of IL6 (G) and TNFα (H) in liver sections. I) Quantification of iNOS^+^F4/80^+^ cells. J) Mice were given 3 mg kg^−1^ TCP or vehicle (corn oil) 48 h after surgery, following treatment with 10 mg kg^−1^ LPS via intravenous injection. Mice were sacrificed at the indicated time point after LPS treatment. K) Representative morphological photographs of the mouse liver. L) Liver‐to‐body weight ratio. M, N) Representative images and quantification of Ki67 immunochemical labeling. O) Serum ALT and AST levels. P,Q) Representative images and quantification of IL6 (P) and TNFα (Q) staining. Data are means ± SD. ^*^ *p *< 0.05, ^**^ *p *< 0.01, ^***^ *p *< 0.001, and ^****^ *p *< 0.0001. Statistical analyses were performed using one‐way ANOVA with Dunnett's multiple comparisons test, or unpaired Student's *t*‐test.

As a broad range of clinical agents, including DEX, phenytoin (PTN), resveratrol (RES), meclizine (MCZ), and diallyl sulfide (DAS), have been shown to activate CAR, the influence of these drugs on endotoxin‐induced liver injury was explored.^[^ ^18^, ^19^ ^]^ LPS/GaIn‐challenged mice were treated with TCP, DEX, PTN, RES, MCZ, and DAS for 3 days (**Figure** **8**A). The results suggested that all of the above drugs attenuated LPS/GaIn‐induced liver damage to varying degrees; in particular, TCP, DEX, and PTN had better hepatoprotective effects than the other drugs (Figure 8B–F). In addition, CAR agonist treatment resulted in a reduced inflammatory response, including the suppression of IL6 and TNFα production and a decrease in the number of iNOS^+^F4/80^+^ macrophages (Figure 8D–I). These results suggest that drugs with CAR agonistic effects may attenuate endotoxemic liver damage.

Infection and recovery failure after hepatectomy are the major challenges experienced by liver cancer patients.^[^ ^20^ ^]^ CAR activation has previously been reported to promote liver regeneration.^[^ ^21^ ^]^ Therefore, we established an endotoxin‐induced liver injury after partial hepatectomy (PHx) mouse model to explore the effects of CAR activation. The mice subjected to 70% PHx were stimulated with LPS and then treated with vehicle or TCP (Figure 8J). Compared with those of the vehicle‐treated mice, the liver‐to‐body weight ratios of the TCP‐treated mice were significantly greater at 24 and 72 h after LPS treatment (Figure 8K,L). Immunohistochemical staining for a proliferation marker (Ki67) revealed that the number of Ki67^+^ cells in the TCP‐treated group was notably greater than that in the vehicle group at 24 and 72 h (Figure 8M,N). Moreover, the levels of ALT and AST, indices of liver damage, were elevated at 3–6 h and gradually decreased at 6–24 h after LPS injection, whereas the levels of ALT and AST were markedly lower after treatment with TCP (Figure 8O). In addition, immunohistochemical staining revealed that the infiltration of IL6 and TNFα in the livers of the TCP‐treated group was markedly lower than that in the livers of the vehicle group (Figure 8P,Q). These results indicated that CAR activation promoted liver recovery and reduced endotoxin‐induced liver injury after PHx.

## 3 Discussion

Although the importance of CAR in drug and xenobiotic metabolism in hepatocytes is well documented, its involvement in immune responses via liver macrophages has not been previously reported. The present study demonstrated that activation of CAR with agonists suppresses the proinflammatory phenotype in macrophages from healthy donors and macrophage cell lines. We revealed the protective effects of macrophage CAR activation against endotoxin‐induced liver injury by using macrophage‐specific *Car* knockdown and *Tlr4* knockout mice. Specifically, CAR activation in macrophages inhibited the TLR4 signaling pathway, resulting in reduced inflammatory gene expression and cytokine release. This ultimately mitigated hepatocyte death and liver damage in response to LPS challenge. In summary, our findings provide new insights into the relationship between CAR activation and immunological regulation, which could be important for the intervention of inflammatory liver diseases.

Increasing evidence has revealed that the systemic inflammatory response is closely linked to the progression and severity of acute liver failure.^[^ ^22^ ^]^ Macrophages are central regulators of homeostasis and inflammation in liver disease.^[^ ^23^ ^]^ Additionally, some nuclear receptors have been shown to play a protective role by modulating the behavior of macrophages. The activation of peroxisome proliferator‐activated receptor gamma promotes macrophage polarization to the M2 phenotype and improves insulin sensitivity;^[^ ^24^ ^]^ liver X receptor can exert anti‐atherosclerosis effects by promoting cholesterol clearance by macrophages.^[^ ^25^ ^]^ In the present study, CAR was found to be abundant in both human and murine macrophages, suggesting that CAR is a potential target for modulating macrophage function. The inhibitory effect of CAR on LPS‐driven inflammation disappeared when CAR was specifically knocked out or knocked down in macrophages in vivo or in vitro. These findings suggest that CAR in macrophages is essential for the observed protective effect of CAR against endotoxemic liver injury.

TLR4 is the major receptor for LPS and is critical for endotoxin‐induced liver injury.^[^ ^26^ ^]^ Upon endotoxin stimulation, TLR4/NFκB signaling rapidly triggers a potent innate immune response, resulting in the expression and release of a large variety of inflammatory mediators.^[^ ^20^ ^]^ Thus, targeting the TLR4‐mediated NFκB pathway may be a way to alleviate endotoxin‐induced liver failure. By using commercially available CAR‐specific activators, we verified that CAR interacted with *Tlr4* and that activated CAR suppressed LPS‐induced M1‐type macrophages and repressed the activation of inflammatory cytokines, such as *Il6*, *Il1β*, *Tnfα*, *Ccl2*, and *Ccl3*. In *Tlr4* ^−/−^ mice, the inflammation induced by LPS was partially attenuated, which was consistent with the findings of previous studies.^[^ ^26^ ^]^ In contrast, CAR activation did not exert hepatoprotective effects or inhibit LPS‐driven inflammation in LPS‐treated *Tlr4* ^−/−^ mice, suggesting that the protective effect of CAR is mainly TLR4‐dependent.

Prior to the discovery of its anti‐inflammatory effects, CAR was primarily known as a metabolic nuclear receptor. CAR regulates hepatic detoxification processes, serving as a ligand‐activated modulator of xenobiotic‐metabolizing enzymes such as CYPs, conjugating enzymes, and transporters.^[^ ^14^ ^]^ During endotoxin‐induced liver injury, impaired drug delivery is attributed to downregulated gene expression of drug‐metabolizing enzymes and transporters.^[^ ^27^, ^28^ ^]^ Previous studies have reported that hepatic *Cyp2b10* and *Ugt1a1* mRNA expression was downregulated after LPS exposure or *C. rodentium* infection.^[^ ^29^, ^30^ ^]^ In the current study, CAR activation induced the expression of CYP2B10, UGT1A1, and SULT2A1 after LPS treatment, suggesting that CAR activation not only inhibited the uncontrolled inflammatory response but also improved metabolism‐related functions during endotoxin challenge.

Here, DEX effectively inhibited polarization toward the M1 phenotype and cytokine secretion in macrophages, even after treatment with LPS, preventing macrophages from skewing toward a proinflammatory profile.^[^ ^31^, ^32^ ^]^ DEX is often used in clinical practice as an adjuvant treatment for endotoxemia. The immunosuppressive effects of DEX are reported to be mediated by the glucocorticoid receptor (GR), and the binding of GR to NFκB prevents NFκB from initiating transcription.^[^ ^33^ ^]^ Indeed, DEX is also a powerful CAR agonist;^[^ ^34^ ^]^ we observed that DEX promoted CAR nuclear translocation and inhibited the inflammatory response induced by LPS, which might suggest that CAR activation contributed to the formidable immunosuppressive effect of DEX. In addition, the current study revealed that clinically available drugs (DEX, PTN, RES, MCZ, and DAS) with CAR‐agonistic effects have anti‐inflammatory effects and hepatoprotective effects against endotoxin‐induced liver injury; the results obtained from DEX, DAS, and RES are consistent with previous findings.^[^ ^35^, ^36^, ^37^ ^]^ Therefore, CAR activation with agonists after sepsis onset may be a promising strategy for the management of endotoxemic liver disease.

In conclusion, the present study revealed a novel immunoregulatory effect of CAR on macrophage‐mediated inflammation. CAR activation could suppress the proinflammatory phenotype of macrophages and effectively protect against endotoxemic liver injury. These findings pave the way for future exploration of the use of CAR agonists to manage inflammation and may unlock new strategies for alleviating inflammatory liver injury.

## 4 Experimental Section

### Healthy Donors and Culture of hMDMs

Eight healthy volunteers (four males and four females, ethical approval number: SYSKY‐2024‐885‐01) were enrolled in the study. Human monocytes were collected from the blood of the donors using an extraction kit (Solarbio, Beijing, China) and treated with granulocyte macrophage‐colony stimulating factor (GM‐CSF, 100 ng mL^−1^, ABclonal, Wuhan, China) for 7 days.^[^ ^38^ ^]^ After differentiation, hMDMs were cultured in RPMI 1640 medium (Invitrogen, Carlsbad, USA) supplemented with 20% FBS (ZETA Life, California, USA) and 1% antibiotics. The macrophages were preincubated with 1 µM CITCO (MedChemExpress, New Jersey, USA) or 1 µM DEX (Aladdin Biotechnology, Shanghai, China) for 24 h and then coincubated with LPS (1 µg mL^−1^, Aladdin Biotechnology, Shanghai, China) or PBS for an additional 24 h. Experiments were performed in mycoplasma‐free cells.

### Animal Experiments

Male C57BL/6J mice (8–10 weeks) were purchased from Zhuhai BesTest Bio‐Tech Co. (Zhuhai, China). *Tlr4* ^−/−^ mice (8–10 weeks) were obtained from The Jackson Laboratory (Maine, USA). All the mice were housed and cared for in a standard facility located within the Laboratory Animal Center of Sun Yat‐Sen University (Guangzhou, China) in a pathogen‐free system, which provides sterile isolator cages with fresh food, water, and bedding weekly. The studies were conducted following the recommendations of the Sun Yat‐Sen University Institutional Animal Care and Use Committee (ethical approval number: SYSU‐IACUC‐2023‐000483). All animal experiments were conducted with random assignment.

### Animal Experiments—LPS/GaIn Treatment

Mice were injected intraperitoneally with vehicle (corn oil, Sigma, St. Louis, USA) or 3 mg kg^−1^ mouse‐specific CAR agonist TCP (Aladdin Biotechnology, Shanghai, China) once daily for 5 days. Two hours after the last injection of TCP, the mice were intraperitoneally injected with saline or LPS/GaIn (100 µg kg^−1^ and 700 mg kg^−1^, respectively, Aladdin Biotechnology, Shanghai, China). Blood samples and liver tissues were harvested 6 h after LPS/GaIn treatment.

### Animal Experiments—Macrophage‐Specific Car Interfering

To generate macrophage‐specific‐*Car* knockdown mice, AAV8 viral vectors carrying the *F4/80* promoter, *Car* shRNA, and the ZsGreen reporter gene were used. Briefly, the mice received an intravenous injection of AAV8‐*F4/80*‐sh*Car* for macrophage‐specific *Car* knockdown or AAV8‐*F4/80*‐sh*Con* as the negative control (1.0 × 10^12^ genome copies per mouse; Hanbio, Shanghai, China). After 4 weeks of gene silencing, 3 mg kg^−1^ TCP was injected intraperitoneally for 5 consecutive days, and 100 µg kg^−1^ LPS and 700 mg kg^−1^ GaIn were injected intraperitoneally 2 h after the last dose of TCP. Blood samples and liver tissues were harvested 6 h after LPS/GaIn treatment.

### Animal Experiments—Tlr4^−/−^ Mouse Experiments

WT or *Tlr4* ^−/−^ mice were injected intraperitoneally with 3 mg kg^−1^ TCP or vehicle once daily for 5 days. Two hours after the last injection of TCP, the mice were given saline or LPS/GaIn (100 µg kg^−1^ and 700 mg kg^−1^, respectively) intraperitoneally. Blood samples and liver tissues were harvested 6 h after LPS/GaIn treatment.

### Animal Experiments—CAR Agonist Treatment

Mice were injected intraperitoneally with 3 mg kg^−1^ TCP or 50 mg kg^−1^ DEX, 50 mg kg^−1^ PTN, 100 mg kg^−1^ RES, 100 mg kg^−1^ MCZ, 100 mg kg^−1^ DAS, or vehicle (corn oil) once daily for 3 days. Two hours after the last injection of TCP, the mice received saline or LPS/GaIn (100 µg kg^−1^ and 700 mg kg^−1^, respectively) intraperitoneally. Blood samples and liver tissues were harvested 6 h after LPS/GaIn treatment.

### Animal Experiments—PHx with LPS Treatment

The model was established according to a previously described method.^[^ ^20^ ^]^ Briefly, the mice subjected to 70% PHx surgery were injected intraperitoneally with 3 mg kg^−1^ TCP or corn oil 48 h after PHx. Two hours after TCP injection, the mice were injected with saline or 10 mg kg^−1^ LPS intravenously and sacrificed at 0, 1, 3, 6, 24, and 72 h after LPS administration.

### Hepatocyte and Kupffer Cell Isolation

Primary HCs and KCs were isolated from male C57BL/6J mice (8–10 weeks) by using gradient centrifugation.^[^ ^18^ ^]^ The liver was perfused with calcium and magnesium‐free HBSS containing 0.5 mM EGTA, and DMEM containing 0.075% type IV collagenase (Yuanye Bio‐Technology, Shanghai, China), chopped finely, and digested at 37 °C for 20 min. The cell suspensions were filtered through a 150 µm mesh. HCs were gained from the sediment after centrifugation at 50 × g for 5 min three times. The supernatant was then centrifuged at 500 × g for 15 min to precipitate nonparenchymal cells. The nonparenchymal cells were resuspended in Percoll working solution, centrifuged at 800 × g for 15 min at room temperature, and the KCs were collected from the deposit.

### Cell Culture and Treatment—BMDM Treatment

BMDMs were isolated and differentiated according to previous protocols.^[^ ^39^ ^]^ Briefly, BMDMs were collected from the tibia and femur of C57BL/6J mice. Cells were cultured in DMEM (Invitrogen, Carlsbad, USA) supplemented with 20% FBS and 1% antibiotics and differentiated with 25 ng mL^−1^ M‐CSF (ABclonal, Wuhan, China) for 7 days. BMDMs were preincubated with DMSO or 5 µM TCP for 24 h, and then were coincubated with 1 µg mL^−1^ LPS or PBS for 24 h.

### Cell Culture and Treatment—RAW264.7 Cell Treatment

Murine macrophage RAW264.7 cells were obtained from Cellcook (Guangzhou, China). Cells were cultured in DMEM supplemented with 12% FBS and 1% antibiotics. RAW264.7 cells were preincubated with DMSO or 5 µM TCP for 24 h, and then were coincubated with 1 µg mL^−1^ LPS or PBS for 24 h.

### Cell Culture and Treatment—THP‐1 Cell Treatment

Human macrophage THP‐1 cells were obtained from Procell (Wuhan, China). Cells were cultured in RPMI 1640 medium supplemented with 12% FBS, 0.05 mM β‐mercaptoethanol (Procell, Wuhan, China), and 1% antibiotics, and differentiated with 320 nM phorbol 12‐myristate 13‐acetate (PMA, Beyotime, Shanghai, China) for 24 h.^[^ ^40^ ^]^ THP‐1 cells were preincubated with DMSO or 1 µM CITCO for 24 h, and then were coincubated with 100 ng mL^−1^ LPS and 20 ng mL^−1^ IFNγ or PBS for 24 h.

### Cell Culture and Treatment—Coculture System

RAW264.7 and AML12 cells were used to observe the interaction between macrophages and hepatocytes. RAW264.7 cells were seeded in the upper chamber of the plate, and were preincubated with DMSO or 5 µM TCP for 24 h, and then were coincubated with 1 µg mL^−1^ LPS or PBS for 24 h. RAW264.7 were further cocultured with AML12 (bottom chamber) for 24 h in the coculture group; AML12 cells alone were preincubated with DMSO or 5 µM TCP for 24 h, and then were coincubated with 1 µg mL^−1^ LPS or PBS for 24 h.

### Serological Assessments of The Liver Function and Cytokines

The collected blood samples were centrifuged at 3500 rpm for 15 min at 4 °C to obtain the serum. The levels of ALT and AST were analyzed by URIT 8021A automated biochemical analyzer (URIT Medical Electronic, Guilin, China).

Serum TNFα and IL6 were measured using an ELISA kit according to the manufacturer's instructions (Elk biotechnology, Wuhan, China). The levels of TNFα and IL6 were analyzed by the microplate reader (Thermo Scientific, Rockford, USA).

### Histopathological Analysis and TUNEL Assay

Tissues were fixed with 4% paraformaldehyde and embedded in paraffin, and then were cut into 4 µm sections. The deparaffinized and rehydrated paraffin sections were stained with hematoxylin and eosin (Servicebio, Wuhan, China). Images were captured using the EVOS M7000 Imaging Systems (Thermo Scientific, Rockford, USA).

TUNEL staining was used for nuclear DNA fragmentation of apoptotic or necrotic cells (Beyotime, Shanghai, China). Images were captured using the FV3000 confocal microscope (Olympus, Tokyo, Japan).

### Immunohistochemistry and Immunofluorescence Staining

The deparaffinized and rehydrated paraffin sections were heated in citric acid buffer for 30 min. Goat or donkey serum was used to block the non‐specific binding sites for 2 h at room temperature. Sections were incubated with primary antibody at 4 °C overnight and secondary antibody for 1–2 h at room temperature. DAPI for immunofluorescence and hematoxylin for immunohistochemistry were used to counterstain nuclei.

Cells were fixed in the fixative solution (Beyotime, Shanghai, China) for 15 min, permeabilized with 0.1% Triton X‐100 solution for 10 min, incubated with primary antibody at 4 °C overnight. The fluorescent secondary antibody was incubated for 1 h at room temperature. Cellular nuclei were counterstained with DAPI.

Stained sections or cell dishes were photographed using the EVOS M7000 imaging systems (Thermo Scientific, Rockford, USA) or the FV3000 confocal microscope (Olympus, Tokyo, Japan). The rabbit anti‐F4/80 antibody was purchased from Cell Signaling Technology (Danvers, USA); the rat anti‐CLEC4F antibody was purchased from R&D Systems (Minneapolis, USA); the rabbit anti‐CAR, the mouse anti‐F4/80, anti‐IL6, anti‐iNOS, and anti‐CD86 antibodies were purchased from Santa Cruz Biotechnology (California, USA) and the rabbit anti‐TNFα antibody was obtained from ABclonal (Wuhan, China); and the rabbit anti‐CAR antibody was purchased from Proteintech (Wuhan, China). The anti‐mouse IgG Alexa Fluor 488 and anti‐rabbit IgG Alexa Fluor 647 secondary antibodies were obtained from Cell Signaling Technology (Danvers, USA), and the rabbit anti‐Ki67, anti‐CD68, and anti‐rat IgG Alexa Fluor 488 antibodies were purchased from Abcam (Cambridge, UK).

### Quantitative Real‐Time Polymerase Chain Reaction Analysis

Total RNA was extracted from liver tissue or cells using a Trizol reagent (Invitrogen, New York, USA). RNA was reverse‐transcribed using a PrimeScript RT reagent kit (Accurate Biology, Changsha, China). The cDNA amplification was performed in an ABI‐Prism 7500 Sequence Detection System (Applied Biosystems, Foster City, USA) using the SYBR Green pro‐Taq HS qPCR kit (Accurate Biology, Changsha, China). The relative expression of the target genes was normalized to the housekeeping gene. Primers are listed in Tables S1 and S2 (Supporting Information).

### RNA Sequencing Analysis

Total RNA was extracted from LPS/GaIn‐treated and LPS/GaIn + TCP‐treated mice. RNA sequencing and bioinformatics analysis were conducted in LC‐Bio Technology as previously described.^[^ ^41^ ^]^ High‐throughput sequencing was performed on an Illumina Novaseq 6000 (LC‐Bio Technology Co., Ltd., Hangzhou, China) following the vendor's recommendations.

### Western Blotting Analysis

Livers or cells were homogenized with RIPA lysis buffer containing PMSF and phosphatase inhibitors (Biocolors, Shanghai, China). Nuclear proteins were extracted using a nuclear/cytosol extraction kit purchased from Sangon Tech (Shanghai, China). Proteins were separated by electrophoretically on polyacrylamide gels (8%–12% SDS gels) and then transferred to polyvinylidene dichloride membranes (0.2 or 0.45 µm, Millipore, Bedford, USA) or nitrocellulose membrane (BioTrace NT, PALL, Washington, USA). Membranes were blocked in 5% BSA or 5% nonfat milk for 2 h at room temperature, incubated with primary antibodies overnight at 4 °C, and then secondary antibodies for 2 h at room temperature. The membranes were probed with an electrochemiluminescence detection kit (Millipore, Bedford, USA) and imaged using a Tanon 5200 auto‐imaging system (Shanghai, China).

Mouse polyclonal anti‐TLR4, anti‐p‐NFκB p65, anti‐NFκB p65, anti‐CYP2B10, and anti‐SULT2A1 antibodies were purchased from Santa Cruz Biotechnology (California, USA). Anti‐p‐IκB antibody was purchased from ABclonal (Wuhan, China). Anti‐IκB, anti‐GAPDH, and anti‐β‐ACTIN antibodies were purchased from Cell Signaling Technology (Danvers, USA). The mouse monoclonal anti‐LMNB1 antibody was purchased from Sangon Biotechnology (Shanghai, China). The rabbit anti‐p‐IKK antibody was acquired from Beyotime Biotechnology (Shanghai, China). The rabbit anti‐UGT1A1 antibody was purchased from Signalway Antibody (Nanjing, China).

### Flow Cytometric Assessment

Single cell suspensions of 1 × 10^7^ cells mL^−1^ were washed with PBS. RAW264.7 cells and BMDMs were incubated with the anti‐FcR antibody CD16/32 (Elabscience, Wuhan, China) for 10 min at room temperature to block nonspecific binding. Cells were stained with anti‐CD86‐PE (Elabscience, Wuhan, China) or anti‐iNOS‐FITC (Santa Cruz Biotechnology, California, USA) at 4 °C for 30 min. hMDMs and THP‐1 cells were blocked with the purified anti‐human CD16 antibody (Elabscience, Wuhan, China). hMDMs were stained with anti‐CD68‐PE (Santa Cruz Biotechnology, California, USA) and anti‐CD86‐APC (Elabscience, Wuhan, China). THP‐1 cells were stained with anti‐CD86‐APC (Elabscience, Wuhan, China) or anti‐CD80‐APC (Elabscience, Wuhan, China). Flow cytometry analysis was performed using the Beckman FACS flow cytometer (California, USA).

### Nuclear Translocation

RAW264.7 cells were preincubated with DMSO or 5 µM TCP for 24 h, and then were coincubated with 1 µg mL^−1^ LPS or PBS for 2 h. THP‐1 cells were preincubated with DMSO or 1 µM CITCO for 24 h, and then were coincubated with 100 ng mL^−1^ LPS and 20 ng mL^−1^ IFNγ or PBS for 2 h. The cells were fixed in the fixative solution (Beyotime, Shanghai, China) for 15 min, permeabilized by 0.1% Triton X‐100 solution for 10 min, and incubated with anti‐NFκB p65 antibody (Santa Cruz Biotechnology, California, USA) at 4 °C overnight. The fluorescent secondary antibody (anti‐mouse IgG Alexa Fluor 488, Cell Signaling Technology, Danvers, USA) was incubated with RAW264.7 or THP‐1 cells for 1 h at room temperature. Cellular nuclei were counterstained with DAPI. Images were acquired using the FV3000 confocal microscope (Olympus, Tokyo, Japan).

### Electrophoretic Mobility Shift Assay

Nuclear proteins from RAW264.7 cells were prepared using a nuclear/cytosol extraction kit (Sangon Tech, Shanghai, China). Synthetic complementary NFκB (5′‐AGT TGA GGG GAC TTT CCC AGG C‐3′) binding oligonucleotides with 3′‐ biotinylation were obtained from Sangon Biotechnology (Shanghai, China). The protein‐DNA reactions were conducted for 20 min at room temperature, and the molecular interaction was analyzed by electrophoresis on a nondenaturing 4% polyacrylamide gel, followed by autoradiography according to the instructions for the Chemiluminescent EMSA Kit (Beyotime Biotechnology, Shanghai, China).

### RNA Interference

*Car* and *Tlr4* siRNAs were purchased from RiboBio (Guangzhou, China). siRNAs were transfected into RAW264.7 cells for 24 h using the Lipofectamine 2000 transfection reagent (Vazyme, Nanjing, China), followed by treatment as described above. The sequences of the siRNAs are listed in Table S3 (Supporting Information).

### Actinomycin D Chase

RAW264.7 cells were plated in 6‐well plates and treated with 1 µg mL^−1^ LPS for 24 h. After LPS treatment, 20 µg mL^−1^ ActD (KKL MED, Ashland, USA) with or without 5 µM TCP was added to each well, and RNA was extracted at different time points after ActD exposure.

### RNA Immunoprecipitation

The RIP Kit (Genecreate, Wuhan, China) was used to perform the RIP assay. The magnetic beads were resuspended and washed with the RIP wash buffer. Anti‐CAR (Santa Cruz Biotechnology, California, USA) or anti‐IgG (Genecreate, Wuhan, China) antibodies were used for the assay. The RNA bound to the magnetic beads was examined by qRT‐PCR.

### RNA Pull‐Down Assay

An RNA pull‐down kit (Genecreate, Wuhan, China) was used to perform the RNA pull‐down assay. Briefly, a biotin‐labeled probe targeting the CDS and each domain of *Tlr4* was synthesized (Sangon Tech, Shanghai, China), and mouse livers were harvested and lysed in IP lysis buffer on ice for 30 min. The nucleic acid‐compatible streptavidin magnetic beads (Genecreate, Wuhan, China) were incubated with the biotin‐labeled probe for 2 h to generate probe‐combined beads. The liver lysates were incubated with the beads for pull‐down CAR for 2 h. The enriched proteins were eluted and examined by Western blotting analysis. Sequences of the probes are listed in Table S4 (Supporting Information).

### Statistical Analysis

Paired data were analyzed using a paired *t*‐test. An unpaired Student's *t*‐test was used for statistical analysis of unpaired data. Differences in a single variable across multiple groups were evaluated via one‐way analysis of variance (ANOVA) using GraphPad Prism 9.5 (GraphPad Software Inc., San Diego, USA). Data are means ± SD. *p*‐values *<* 0.05 were considered to indicate significance. The TOC figure was created with Figdraw 2.0.

### Ethic Approval

This study involved human samples that were approved by Sun Yat‐Sen Memorial Hospital (ethical approval number: SYSKY‐2024‐885‐01), and all experiments were conducted according to the national and institutional guidelines. Mouse experiments were performed in accordance with the ARRIVE guidelines and approved by Sun Yat‐Sen University Institutional Animal Care and Use Committee (ethical approval number: SYSU‐IACUC‐2023‐000483)

## Conflict of Interest

The authors declare no conflict of interest.

## Author Contributions

R.C., T.Z., and Y.W. contributed equally to this work. Y.J. conceptualized the project. Y.J. and R.C. designed experiments. R.C., T.Z., Y.W., S.S., Y.L., M.H., Y.Z., S.H., S.W., and Y.W. performed experiments. H.P. and M.H. provided valuable intellectual input throughout. R.C., T.Z., and Y.J. drafted the manuscript. Y.J. and M.H. supervised the study.

## Supporting information



Supporting Information

## Data Availability

The accession number for the RNA‐seq data is GSE283511. The data that support the findings of this study are available from the corresponding author upon reasonable request.
