# Supplementary material for: Constitutive Androstane Receptor in Macrophages Regulates Toll‐Like Receptor 4‐Mediated Innate Immune Responses Against Endotoxemic Liver Injury
Source: Adv Sci (Weinh). 2025 Aug 18;12(42):e06725. doi: 10.1002/advs.202506725 (PMC12622418; doi:10.1002/advs.202506725)
Supplement: Supplementary file 1 — Supporting Information [file ADVS-12-e06725-s001.docx]

**Supporting Information**

**Constitutive Androstane Receptor in Macrophages Regulates Toll-like Receptor 4-mediated Innate Immune Responses against Endotoxemic Liver Injury**

*Renjie Cao^†^, Tingting Zhao^†^, Ying Wang^†^, Shaofei Song, Yuan Li, Mengling Hou, Yanxin Zhang, Sengpeng Wong, Siqi Wang, Yuran Wang, Hong Peng, Min Huang, Yiming Jiang^*^*

R. Cao, T. Zhao, S. Song, Y. Li, M. Hou, Y. Zhang, S. Wong, S. Wang, Y. Wang, M. Huang, Y. Jiang

Guangdong Provincial Key Laboratory of New Drug Design and Evaluation, School of Pharmaceutical Sciences, Sun Yat-Sen University

Institute of Clinical Pharmacology, Sun Yat-Sen University

Guangzhou 510006, China.

E-mail: jiangym25@mail.sysu.edu.cn (Yiming. Jiang, ORCID: 0000-0002-4894-177X)

Y. Wang

Sun Yat-Sen Memorial Hospital, Sun Yat-Sen University

Guangzhou 510006, China.

H. Peng

Center of Hepato-Pancreato-biliary Surgery, The First Affiliated Hospital of Sun Yat-Sen University

Guangzhou 510006, China.

^†^ These authors contributed equally to this work.

^*^Corresponding author

**Statements:**

Ethic approval
This study involved human samples were approved by Sun Yat-Sen Memorial Hospital (ethical approval number: SYSKY-2024-885-01), and all experiments were conducted according to the national and institutional guidelines. Mouse experiments were performed in accordance with the ARRIVE guidelines and approved by Sun Yat-Sen University Institutional Animal Care and Use Committee (ethical approval number: SYSU-IACUC-2023-000483).

Conflict of interest

The authors declare no conflict of interest.

1. **Supplementary Figures**


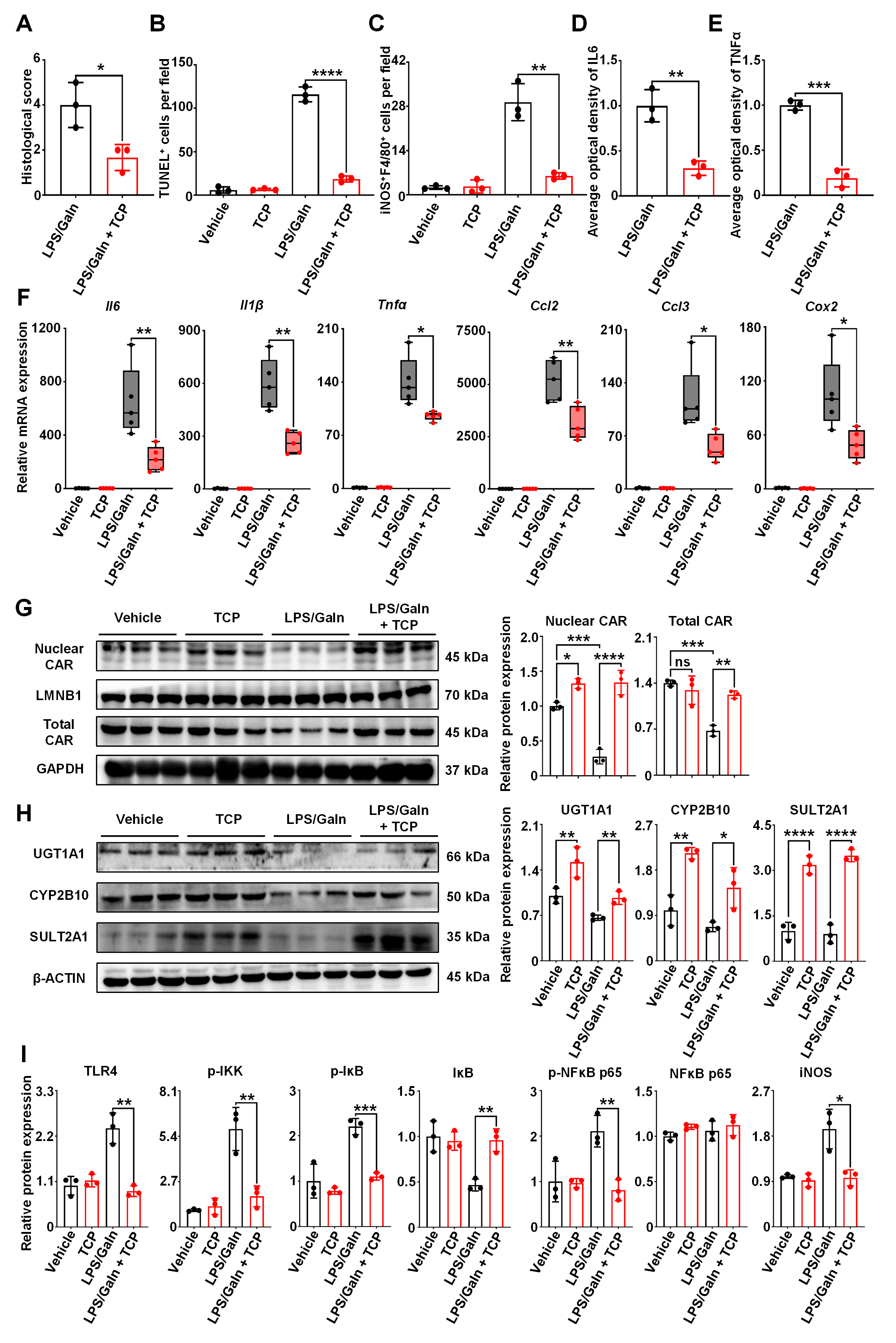


**Figure S1.** CAR activation protects against LPS/GaIn-driven liver injury and alleviates liver inflammation. A) Histological score of the H&E. B) Quantification of TUNEL^+^ cells in liver sections. C) Quantification of iNOS^+^F4/80^+^ cells in liver section. D, E) Quantification of IL6 (D) and TNFα (E) in liver section. F) qRT-PCR of the inflammatory genes (*Il6, Il1β, Tnfα, Ccl2*, *Ccl3*, and *Cox2*). G) Western blotting and quantification of nuclear and total CAR expression in liver. H) Western blotting and quantification of the expression of CAR downstream proteins after TCP treatment. I) Quantification of TLR4/NFκB pathway protein expression after TCP treatment. Data are means ± SD. ^*^*P* < 0.05, ^**^*P* < 0.01, ^***^*P* < 0.001, and ^****^*P* < 0.0001. Statistical analyses were performed using unpaired Student’s *t*-test, or one-way ANOVA with Tukey's multiple comparisons test.


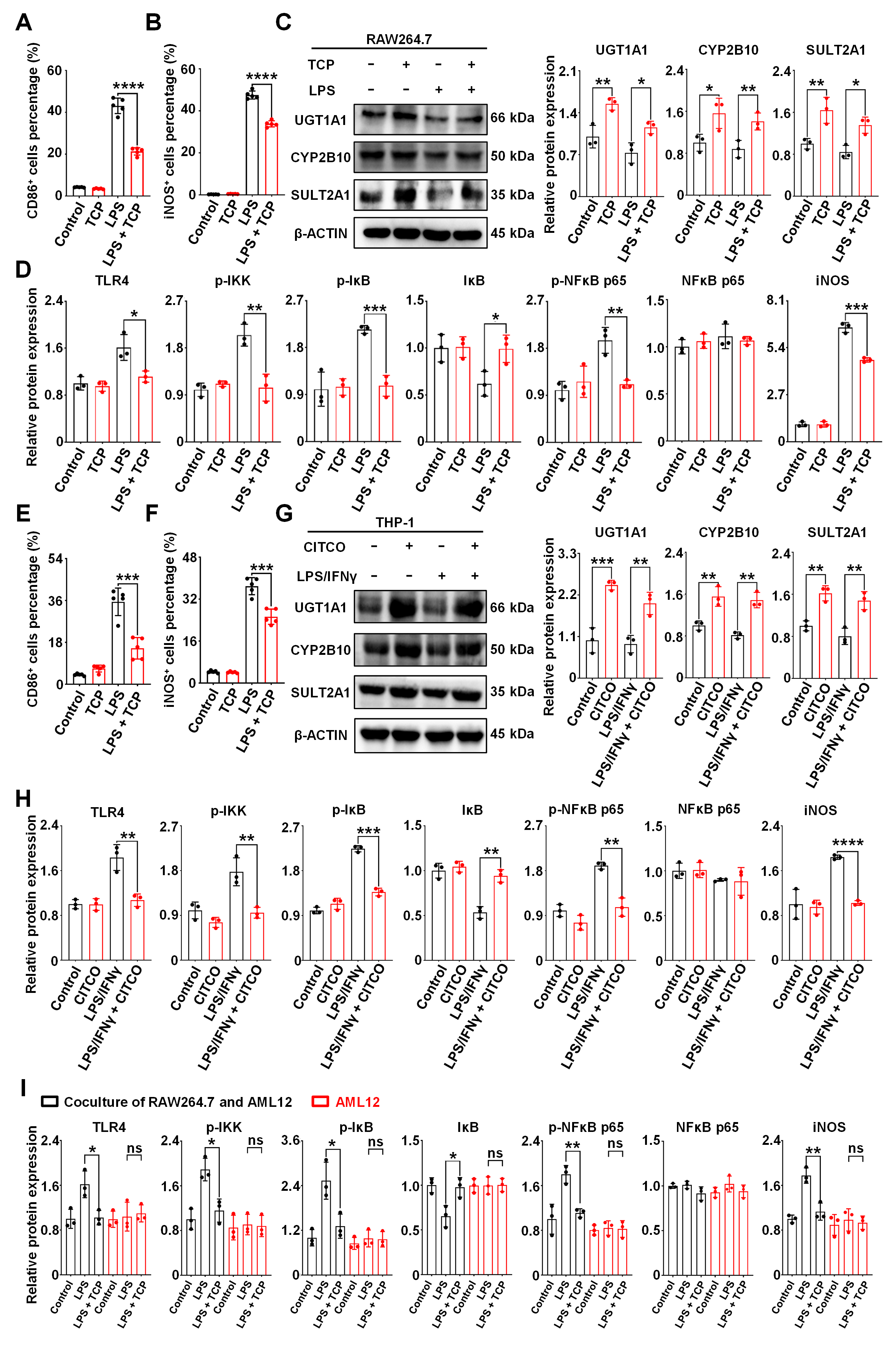


**Figure S2.** Effects of CAR activation in BMDMs, RAW264.7 and THP-1 cells. A, B) Flow cytometric proportion of CD86^+^ (A) and iNOS^+^ (B) RAW264.7 incubated with TCP. C) Western blotting and quantification of the expression of CAR downstream proteins after TCP treatment. D) Quantification of TLR4/NFκB pathway protein expression in RAW264.7 cells. E, F) Flow cytometric proportion of CD86^+^ (E) and iNOS^+^ (F) BMDMs incubated with TCP. G) Western blotting and quantification of the expression of CAR downstream proteins in THP-1 cells after CITCO treatment. H) Quantification of TLR4/NFκB pathway protein expression in THP-1 cells. I) Quantification of TLR4/NFκB pathway protein expression in RAW264.7 and AML12 coculture system. Data are means ± SD. ^*^*P* < 0.05, ^**^*P* < 0.01, ^***^*P* < 0.001, and ^****^*P* < 0.0001. ns: not significant. Statistical analyses were performed using unpaired Student’s *t*-test, or one-way ANOVA with Tukey's multiple comparisons test.


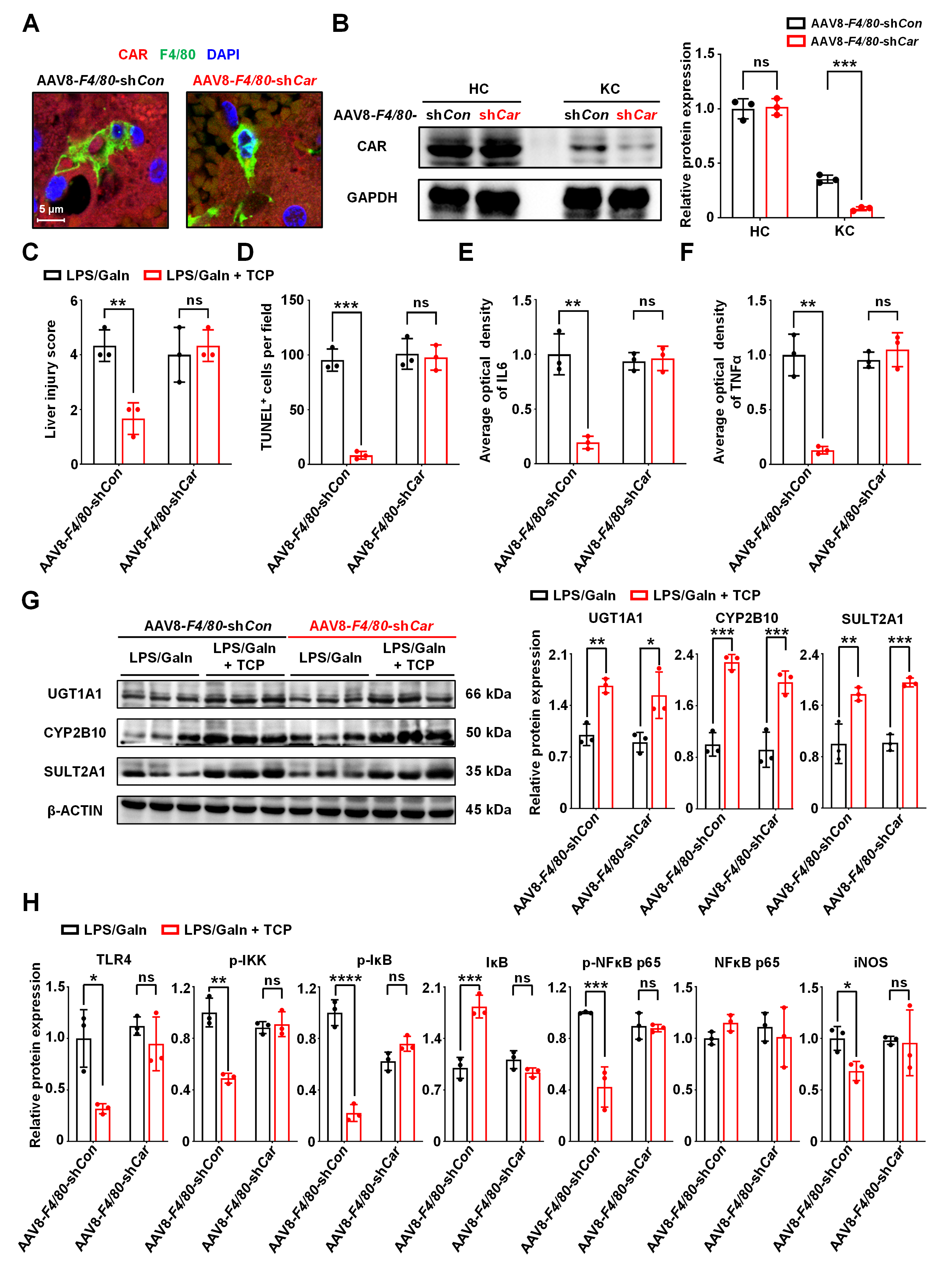


**Figure S3.** Effects of CAR activation in AAV8-*F4/80*-sh*Con* and AAV8-*F4/80*-sh*Car* mice. A) Immunofluorescence staining of CAR (red) and F4/80 (green) of liver sections. B) Western blotting and quantification of CAR expression in HC and KC in AAV8-*F4/80*-sh*Con* and AAV8-*F4/80*-sh*Car* mice. C) Histological score of the H&E. D) Quantification of TUNEL^+^ cells in liver sections. E, F) Quantification of IL6 (E) and TNFα (F) in liver sections. G) Western blotting and quantification of the expression of CAR downstream proteins after TCP treatment. H) Quantification of TLR4/NFκB pathway protein expression after TCP treatment. Data are means ± SD. ^*^*P* < 0.05, ^**^*P* < 0.01, ^***^*P* < 0.001, and ^****^*P* < 0.0001. ns: not significant. Statistical analyses were performed using an unpaired Student’s *t*-test.


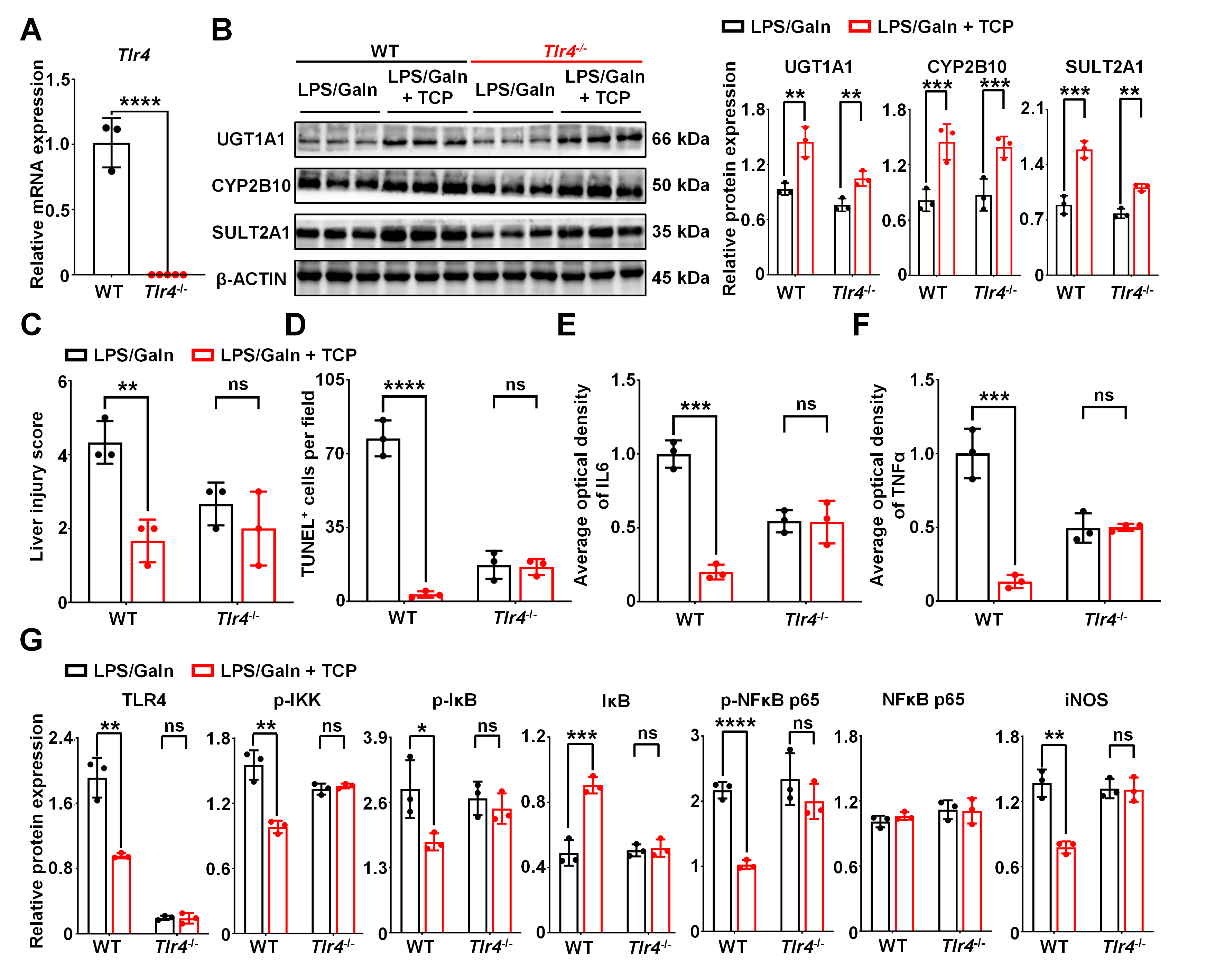


**Figure S4.** Effects of CAR activation in WT and *Tlr4*^-/-^ mice. A) The expression of the *Tlr4* in WT and *Tlr4*^-/-^ mice. B) Western blotting and quantification of the expression of CAR downstream proteins after TCP treatment. C) Histological score of the H&E. D) Quantification of TUNEL^+^ cells in liver sections. E, F) Quantification of IL6 (E) and TNFα (F) in liver sections. G) Quantification of TLR4/NFκB pathway protein expression after TCP treatment. Data are means ± SD. ^*^*P* < 0.05, ^**^*P* < 0.01, ^***^*P* < 0.001, and ^****^*P* < 0.0001. ns: not significant. Statistical analyses were performed using an unpaired Student’s *t*-test.


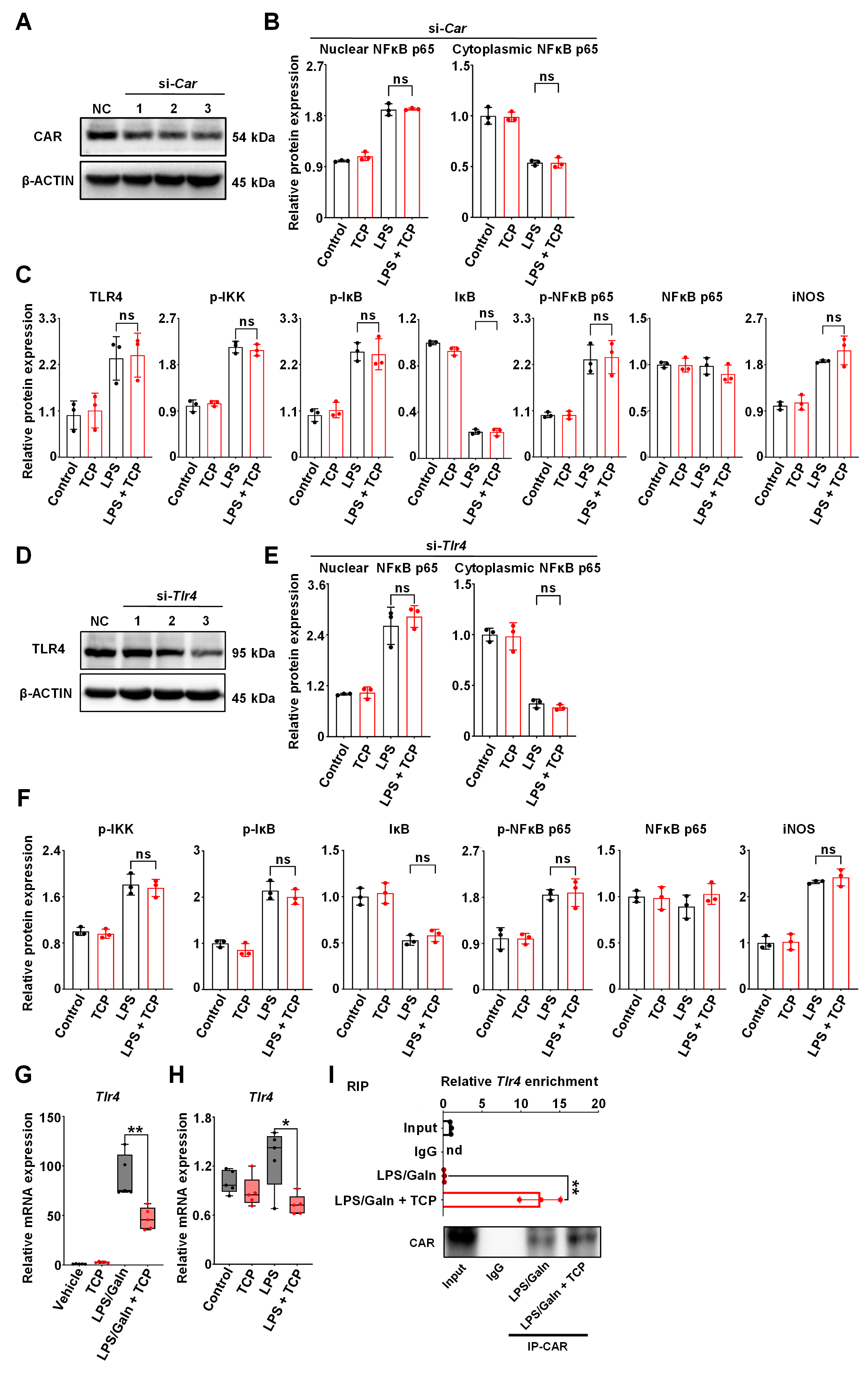


**Figure S5.** Effects of CAR activation after *Car* or *Tlr4* silencing. A) Western blotting analysis of the interference efficiency of si-*Car* on CAR protein expression. B) Quantification of nuclear NFκB p65 and cytoplasmic NFκB p65 after *Car* knockdown in RAW264.7 cells. C) Quantification of TLR4/NFκB pathway protein expression after *Car* knockdown*.* D) Western blotting analysis of the interference efficiency of si-*Tlr4* on TLR4 protein expression. E) Quantification of nuclear NFκB p65 and cytoplasmic NFκB p65 in RAW264.7 after si-*Tlr4*. F) Quantification of the expression of TLR4/NFκB downstream proteins in RAW264.7 after *Tlr4* knockdown in RAW264.7 cells*.* G, H) The gene expression of the *Tlr4* in mice livers (G) or RAW264.7 cells (H). I) RIP assay for CAR and *Tlr4* in LPS/GaIn and LPS/GaIn + TCP group mice. Normalized data were shown as relative fold enrichment to the input. nd: not determined. ^*^*P* < 0.05, and ^**^*P* < 0.01. Data are means ± SD. ns: not significant. Statistical analyses were performed using an unpaired Student’s *t*-test.

1. **Supplementary Tables**

**Table S1.** List of mouse primers used in this study.

| **Gene** | **Forward (5’-3’)** | **Reverse (5’-3’)** |
| --- | --- | --- |
| *Tlr4* | TCCCTGCATAGAGGTAGTTCC | TCAAGGGGTTGAAGCTCAGA |
| *Il6* | GAACAACGATGATGCACTTGC | TCCAGGTAGCTATGGTACTCC |
| *Il1β* | CCAAACCTCTTCGAGGCACA | GCTGCTTCAGACACTTGAGC |
| *Tnfα* | AATGGCCTCCCTCTCATCAGTT | CCACTTGGTGGTTTGCTACGA |
| *Ccl2* | CACTCACCTGCTGCTACTCA | GCTTGGTGACAAAAACTACAGC |
| *Ccl3* | TGAGAGTCTTGGAGGCAGCGA | TGTGGCTACTTGGCAGCAAACA |
| *Cox2* | TGAGTACCGCAAACGCTTCT | CAGCCATTTCCTTCTCTCCTGT |
| *18s* | CCTGGATACCGCAGCTAGGA | GCGGCGCAATACGAATGCCCC |
| *β-actin* | CACTGTCGAGTCGCGTCC | TCATCCATGGCGAACTGGTG |

**Table S2.** List of human primers used in this study.

| **Gene** | **Forward (5’-3’)** | **Reverse (5’-3’)** |
| --- | --- | --- |
| *IL6* | ATGCAATAACCACCCCTGAC | GAGGTGCCCATGCTACATTT |
| *IL1β* | ATGATGGCTTATTACAGTGGCAA | GTCGGAGATTCGTAGCTGGA |
| *TNFα* | CCTCTCTCTAATCAGCCCTCTG | GAGGACCTGGGAGTAGATGAG |
| *CCL2* | GATCTCAGTGCAGAGGCTCG | TTTGCTTGTCCAGGTGGTCC |
| *18S* | ATCCCTGAAAAGTTCCAGCA | CCCTCTTGGTGAGGTCAATG |
| *β-ACTIN* | CATGTACGTTGCTATCCAGGC | CTCCTTAATGTCACGCACGAT |

**Table S3.** Sequences for siRNA

| **siRNA** | **Sequence (5’-3’)** |
| --- | --- |
| *Car* siRNA-1 | ACCAGTTTGTGCAGTTCAA |
| *Car* siRNA-2 | GCAGATATCAACACGTTTA |
| *Car* siRNA-3 | GCTATGCTAACACTAGAAA |
| *Tlr4* siRNA-1 | CAATTCTGTTGCTTGTATA |
| *Tlr4* siRNA-2 | CAATCTGACGAACCTAGTA |
| *Tlr4* siRNA-3 | GTCCCTGATGACATTCCTT |

**Table S4.** List of primers used in RNA pull-down.

| **Gene** | **Forward (5’-3’)** | **Reverse (5’-3’)** |
| --- | --- | --- |
| *Tlr4* | ATGATGCCTCCCTGGCTCCT | TCAGGTCCAAGTTGCCGTTTC |
| *1-779* | ATGATGCCTCCCTGGCTCCT | CCCAAGATCAACCGATGGACG |
| *780-1639* | CGTCCATCGGTTGATCTTGGG | GAGTGCTGAGGGAATACAGC |
| *1640-2508* | GCTGTATTCCCTCAGCACTC | TCAGGTCCAAGTTGCCGTTTC |
